# Lymphoid Organ Architecture and Hematopoiesis Disruption in Spinal Muscular Atrophy: Therapeutic Rescue by SMN Restoration

**DOI:** 10.3390/ijms27031274

**Published:** 2026-01-27

**Authors:** Paula Guillamón, Georg Lindner, Joel Guillen, Alaó Gatius, Sílvia Gras, Laura Martínez-España, Lídia Piedrafita, Anaïs Panosa, Olga Tapia, Conchi Mora, Josep E. Esquerda, Eduardo F. Tizzano, Olga Tarabal, Jordi Calderó

**Affiliations:** 1 Experimental Neuromuscular Pathology Unit, Departament de Medicina Experimental, Facultat de Medicina, Universitat de Lleida, Av. Rovira Roure 80, 25198 Lleida, CAT, Spain; paula.guillamon@udl.cat (P.G.); joelgg66@hotmail.com (J.G.); alao.gatius@udl.cat (A.G.); silvia.gras@udl.cat (S.G.); laura.martinezespana@udl.cat (L.M.-E.); lidia.piedrafita@udl.cat (L.P.); olga.tarabal@udl.cat (O.T.); 2 Institut de Recerca Biomèdica de Lleida—Fundació Dr. Pifarré, IRBLleida, 25198 Lleida, CAT, Spain; anais.panosa@udl.cat (A.P.); conchi.mora@udl.cat (C.M.); josep.esquerda@udl.cat (J.E.E.); 3 Medicine Genetics Group, Vall d’Hebron Research Institute (VHIR), Esfera UAB, 08035 Barcelona, CAT, Spain; georg.lindner17@gmail.com; 4 Servei de Microscòpia i Citometria, Universitat de Lleida, 25198 Lleida, CAT, Spain; 5 Department of Basic Medical Sciences, Institute of Biomedical Technologies (ITB), Universidad de La Laguna (ULL), 38200 Tenerife, CAN, Spain; otapiama@ull.edu.es; 6 Research Group in Immunology and Metabolism (GRIM), Departament de Medicina Experimental, Facultat de Medicina, Universitat de Lleida, 25198 Lleida, CAT, Spain; 7 Neuromuscular Pathology Unit, Fundació Hospital Sant Joan de Déu, 08950 Esplugues de Llobregat, Barcelona, CAT, Spain

**Keywords:** SMA, immune system, SMN∆7 mouse, thymus, spleen, bone marrow, human, SMN-ASO therapy

## Abstract

Spinal muscular atrophy (SMA) is a neuromuscular disorder caused by loss of the *SMN1* gene, reduced levels of SMN protein, and motor neuron degeneration. However, increasing evidence shows that SMA is a multisystemic disease with immune system involvement. We investigated how SMN deficiency affects lymphoid organ development and function using a severe SMA mouse model (SMNΔ7) and postmortem human fetal and postnatal tissues lacking *SMN1* and carrying one or two *SMN2* copies, consistent with type 0–I SMA. Histology, immunostaining, and flow cytometry were used to examine tissue architecture and immune cell composition. SMNΔ7 mice displayed thymus, spleen, and bone marrow abnormalities, including mislocalization of T- and B-cells and expansion of resident macrophages. Bone marrow analysis revealed impaired B-cell development, suggesting intrinsic hematopoietic defects rather than apoptosis. Early treatment with a nusinersen-like antisense oligonucleotide, administered intracerebroventricularly or subcutaneously, restored *SMN2* splicing, improved survival, motor function, and prevented lymphoid pathology. Human SMA samples exhibited similar, though milder, splenic alterations compared to SMNΔ7 mice, while thymic organization remained largely preserved. These findings demonstrate that SMN deficiency disrupts lymphoid organ development through defective bone marrow output and impaired immune cell maturation. Early SMN restoration prevents these abnormalities, highlighting immune dysfunction as a key component of SMA pathology.

## 1. Introduction

Spinal muscular atrophy (SMA) is the most common infantile-onset autosomal recessive neuromuscular disease, with an incidence of approximately 1 in 6000–10,000 live births and a carrier frequency of 1 in 40–70 individuals [1,2]. SMA is caused by homozygous deletions or mutations in the *survival motor neuron 1* (*SMN1*) gene located on chromosome 5q, leading to deficiency of the survival motor neuron (SMN) protein, which is essential for multiple cellular processes, including RNA metabolism, molecular trafficking, cytoskeletal organization, mitochondrial homeostasis, endocytosis, and cell signaling [3,4]. Loss of functional SMN causes degeneration of lower motor neurons (MNs), resulting in progressive muscle weakness and atrophy, paralysis, and in severe cases, respiratory failure and early death [5,6]. Humans possess a nearly identical centromeric copy gene, *SMN2*, which, due to a single nucleotide substitution (c.840C>T), predominantly produces transcripts lacking exon 7, generating a truncated and unstable SMNΔ7 protein [7,8]. The low levels (~10–20%) of functional full-length (FL) SMN protein produced by *SMN2* are insufficient to fully compensate for the lack of *SMN1*, leading to disease. SMA exhibits phenotypic heterogeneity and can be classified based on age of onset and the highest motor abilities achieved, ranging from the most severe (type 0) to the mildest form (type IV). The most common forms are type I (never sit), followed by type II (never walk), and type III (can walk but may lose this ability later in life). The clinical severity and progression rate of SMA inversely correlate with the *SMN2* copy number, which exhibits natural variability across the general population. Patients with fewer *SMN2* copies tend to exhibit more severe disease phenotypes [9,10]. Therefore, one *SMN2* copy generally correlates with type 0, two *SMN2* copies with type I, three *SMN2* copies with type II, and four *SMN2* copies with type III disease. However, this correlation is not absolute, with some exceptions due to different genetic, biological, or still unknown factors [11]. Indeed, additional genes are candidates to be disease modifiers [12].

Although it is well-established that spinal MNs are particularly vulnerable to SMN deficiency, the mechanisms by which reduced SMN levels cause a disease with predominantly neuromuscular involvement remain unclear [13,14]. Additionally, increasing evidence indicates that MN pathology alone cannot fully account for the SMA phenotype. In mouse models of severe SMA, only 20–25% of spinal MNs are lost at end-stages of disease, as determined by exhaustive serial section analyses [15,16], indicating that MN loss is not the main driver of animal demise. In line with this, the selective increase in SMN levels in MNs of SMA mouse models has been reported to result in limited improvements in clinical phenotype and survival [17,18,19]. Similarly, selective *SMN* knockdown in mouse MNs has been shown to result in structural and functional neuromuscular defects, reminiscent of those observed in SMA, but has failed to fully recapitulate the severity of an SMA phenotype [20]. In parallel, defects in other cells and tissues, both within and outside the motor system, have gradually emerged as contributors to the pathogenesis of SMA. Alterations in non-neuronal tissues and organs such as the skeletal muscle, heart, liver, kidney, pancreas, intestine, lung, bone, and immune system, among others, have been described (summarized in [21,22,23,24]). Collectively, these findings highlight the essential role of SMN in peripheral tissues and support the view that SMA should be regarded not merely as an MN or neuromuscular disease, but rather as a multisystem disorder.

There is currently no definitive cure for SMA, but major therapeutic advances have been achieved in recent years. Three SMN-dependent strategies are approved by the Food and Drug Administration (FDA) and the European Medicines Agency (EMA): the antisense oligonucleotide nusinersen (Spinraza^®^, Biogen, Cambridge, MA, USA), the small-molecule splice modulator risdiplam (Evrysdi^®^, Roche, Basel, Switzerland), and the gene replacement therapy onasemnogene abeparvovec (Zolgensma^®^, Novartis Gene Therapies, Bannockburn, IL, USA). All three therapies increase FL-SMN protein levels and, when administered presymptomatically, markedly improve motor function, milestone acquisition, respiratory performance, and survival [25]. As these therapies extend the patient’s lifespan, they are uncovering previously unrecognized multiorgan dysfunctions that were masked by early mortality in severe, untreated patients. The extent to which current treatments correct peripheral pathologies beyond the neuromuscular system remains uncertain, but such manifestations are expected to become increasingly relevant, particularly in patients receiving therapies that primarily target the central nervous system (CNS).

Immune system dysregulation in SMA remains poorly understood. Over a decade ago, we reported for the first time developmental defects in the thymus and spleen of a mouse model of severe SMA [16]. Specifically, we showed that *Smn^−/−^;SMN2^+/+^* mice exhibited marked atrophy of these lymphoid organs, with the thymus showing massive death of cortical thymocytes, particularly at the terminal stages of the disease. Subsequent studies in various SMA models, including *Smn^−/−^;SMN2^tg/0^* (Taiwanese), SMN∆7, and *Smn^2B/−^* mice, have confirmed our findings and revealed disrupted spleen architecture, reduced cellularity, mislocalization of immune cells, and thymic defects in T-cell development [26,27,28]. Collectively, these findings underscore the essential role of SMN in immune system development and suggest that lymphoid organ defects may contribute to disease progression via immunodeficiency and/or neuroinflammation [29]. Therefore, a comprehensive analysis of the immune cell populations associated with lymphoid organs in SMA is essential for understanding the intrinsic consequences of immune dysfunction on the pathogenesis of this disease. In this regard, the bone marrow, which is responsible for B-cell and myeloid cell maturation, remains largely unexplored. Given its critical role in hematopoiesis, research aimed at analyzing potential bone marrow alterations in SMA is crucial for gaining new insights into immune cell development dysregulation and its impact on the disease. Additionally, it is unclear whether current SMN-restoring therapies are sufficient to prevent lymphoid organ pathology and how this may influence disease progression.

In this study, we characterized the histological architecture and cytological alterations of the thymus and spleen in the SMN∆7 mouse model, which recapitulates a severe form of SMA. We further examined changes in lymphocyte and macrophage populations within these organs, as well as their progenitors in the bone marrow. We also performed a histopathological analysis of human samples, which revealed alterations partially overlapping with those observed in the mouse model, providing preliminary validation of these defects in SMA patients. We also evaluated whether a nusinersen-like ASO (SMN-ASO), which mimics the first FDA-approved SMN-dependent drug for SMA treatment, could rescue lymphoid organ and immune cell impairments in SMN∆7 mice, comparing intracerebroventricular (ICV) and subcutaneous (SC) administration routes. Our results reveal that SMA leads to significant structural abnormalities in the spleen, thymus, and bone marrow, with marked dysregulation of immune cell populations. These pathological changes were largely prevented by SMN-ASO treatment, regardless of the delivery route (SC or ICV). This study complements and expands upon previous findings highlighting the critical role of SMN in immune organ development and maturation [16,26,27,28]. Our analysis further underscores the contribution of impaired bone marrow hematopoiesis to immune dysfunction observed in SMA pathology and the therapeutic potential of SMN restoration to prevent these abnormalities.

## 2. Results

### 2.1. Central or Systemic Delivery of SMN-ASO Improves Disease Outcome in SMA Mice

**Figure 1:**
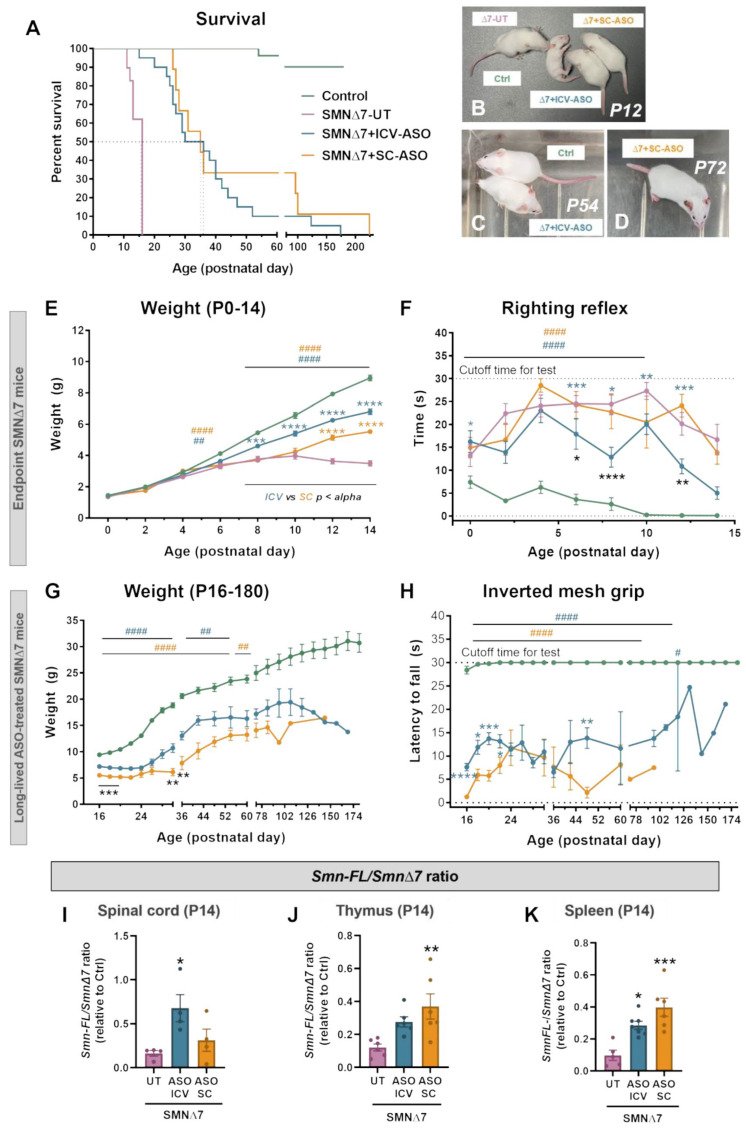
ICV and SC administration of SMN-ASO extends survival, improves motor function, and increases SMN transcript levels in the spinal cord and peripheral lymphoid tissues of SMNΔ7 mice. (**A**) Kaplan–Meier survival curves of control (Ctrl) and SMN∆7 mice either untreated (UT, SMN∆7-UT) or treated with SMN-ASO via intracerebroventricular (ICV) injection at birth (P0, SMN∆7+ICV-ASO) or subcutaneous (SC) injections at P1, P3, P5, and P7 (SMN∆7+SC-ASO). Median survival is indicated by dashed lines. ICV-ASO and SC-ASO treatment significantly extended median survival in SMN∆7 mice to 33 and 35 days, respectively, compared with 14 days in untreated mice (**** *p* < 0.0001, Gehan–Breslow–Wilcoxon test). (**B**–**D**) Representative images of the phenotypic appearance of mice from the four groups at different ages, P1 (**B**), P54 (**C**), and P72 (**D**). Note the differences in tail length between control mice and SMNΔ7 mice treated with SMN-ASO via either ICV or SC administration. (**E**–**H**) Body weight and motor function of the different experimental groups. Body weight was monitored from P0 to P180 (P0–P14 (**E**) and P16–P180 (**G**)). Motor function was assessed by righting reflex (P0–P14 (**F**)) and inverted mesh grip test (P14–P180 (**H**)). Mice were weighed every 2 days until P30, every 6 days from P36 to P60, and every 12 days thereafter. Righting reflex times were recorded every 2 days from P0 to P14; grip latencies were recorded every 2 days from P16 to P30, every 6 days from P36 to P60, and every 12 days thereafter. The cutoff time for both tests was 30 s (upper dashed line). Data are presented as mean ± SEM. Significance: * *p* < 0.05, ** *p* < 0.01, *** *p* < 0.001, **** *p* < 0.0001: SMN∆7-UT vs. SMN∆7+ICV-ASO (blue), SMN∆7-UT vs. SMN∆7+SC-ASO (orange), or SMN∆7+ICV-ASO vs. SMN∆7+SC-ASO (black); # *p* < 0.05, ## *p* < 0.01, #### *p* < 0.0001: Ctrl vs. SMN∆7+ICV-ASO (blue) or Ctrl vs. SMN∆7+SC-ASO (orange). Comparisons were performed using multiple unpaired Student’s *t*-tests; *n* = 9–26 animals per group. Group legends in (**A**) apply to (**E**–**H**). (**I**–**K**) Ratio of Smn full-length (FL) to SmnΔ7 transcripts in the spinal cord (**I**), thymus (**J**), and spleen (**K**) of P14 SMNΔ7 mice, either untreated (UT) or treated with SMN-ASO via ICV (ICV-ASO) or SC (SC-ASO) administration. Data are presented as mean ± SEM and normalized to the ratio observed in control mice (*n* = 4–7 per group). Statistical significance was determined by one-way ANOVA followed by Tukey’s post hoc test. Black asterisks indicate significant differences between UT SMNΔ7 mice and both ICV- and SC-ASO-treated groups (* *p* < 0.05, ** *p* < 0.01, *** *p* < 0.001).

To evaluate the impact of SMN restoration in both the CNS and peripheral tissues, SMNΔ7 (*Smn^−/−^;SMN2^+/+^;SMNΔ7^+/+^*) mice were treated with a nusinersen-like ASO (referred to as SMN-ASO) via either ICV or SC administration, or were left untreated. Survival, body weight, and motor function were assessed and compared with those of control (*Smn^+/+^;SMN2^+/+^;SMNΔ7^+/+^*) animals. Both delivery routes significantly extended survival (median: untreated, 14 days; ICV, 33 days; SC, 35 days) (Figure 1A). Representative phenotypes of different experimental groups at different postnatal ages are shown in Figure 1B–D. SMN-ASO treatment partially restored body weight, with no differences between the delivery route (ICV or SC) used (Figure 1E,G). Motor ability was assessed using the righting reflex test from the postnatal day (P) 0 to P14 and the inverted mesh grip test from P16 onwards. SMN-ASO-treated SMNΔ7 via either ICV or SC administration exhibited significantly faster righting times and longer latencies to fall from the inverted mesh compared with untreated mutants, with slightly better performance in ICV-treated animals. However, neither of the treatments restored motor function to control levels (Figure 1F,H). Overall, SMN-ASO therapy prolongs survival, promotes weight gain, and improves motor performance in SMN∆7 mice independently of the administration route.

### 2.2. Intracerebroventricular or Subcutaneous SMN-ASO Enhances SMN2 Exon 7 Inclusion in CNS and Peripheral Tissues

To evaluate the impact of ICV- and SC-SMN-ASO treatment on exon 7 inclusion in *SMN2* transcripts, we quantified *SMN2* splice variants in spinal cord, thymus, and spleen of P14 SMNΔ7 mice using reverse transcription quantitative polymerase chain reaction (RT-qPCR) with three primer pairs: (1) *hSMN2 ex6/ex6*, targeted exon 6, present in all transcript variants (internal control for normalization); (2) *hSMN2 ex6/ex7*, which selectively amplified transcripts that include exon 7 (*SmnFL* isoform); and (3) *hSMN2 ex5-6/ex6-8*, which selectively amplified transcripts lacking exon 7 (*SmnΔ7* isoform). Exon 7 inclusion ratios (*SmnFL/SmnΔ7*) were calculated and expressed as fold changes relative to controls (Figure 1I–K), providing a quantitative measure of exon 7 inclusion and, by extension, the relative abundance of functional vs. non-functional *SMN* transcripts. Untreated SMNΔ7 mice showed markedly reduced exon 7 inclusion in all tissues compared to controls. In the spinal cord, ICV administration induced the most robust increase in exon 7 inclusion, resulting in a substantially higher proportion of FL transcripts compared to untreated SMNΔ7 animals. Interestingly, although to a lesser extent, SC administration promoted exon 7 inclusion in the spinal cord (Figure 1I). In peripheral lymphoid tissues, both treatment routes enhanced exon 7 inclusion in SMNΔ7 mice. Notably, SC administration appeared slightly more effective than ICV in promoting exon 7 inclusion in both the thymus and spleen (Figure 1J–K).

### 2.3. SMA Mice Display Thymic and Splenic Abnormalities That Are Prevented by SMN-ASO Treatment

**Figure 2:**
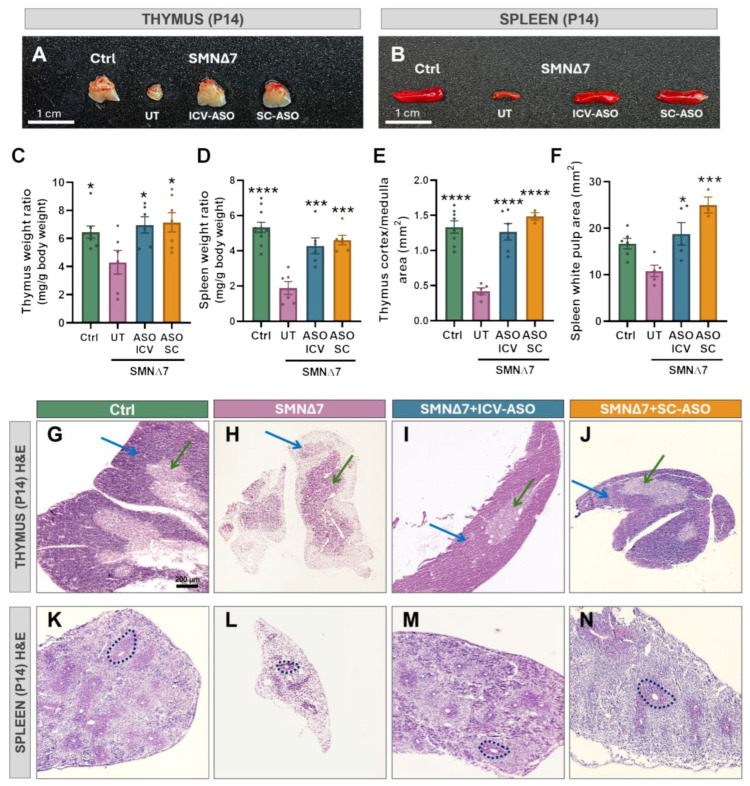
Structural lymphoid organ defects in SMNΔ7 mice are fully or partially prevented by ICV or SC SMN-ASO administration. (**A**,**B**) Macroscopic appearance of the thymus (**A**) and spleen (**B**) from P14 control (Ctrl) and SMNΔ7 mice, either untreated (UT) or treated with SMN-ASO via ICV or SC injections. Scale bars in A and B = 1 cm. (**C**–**F**) Quantification of organ weight and histological parameters in P14 thymus and spleen across the four groups. Thymus (**C**) and spleen (**D**) weights normalized to body weight (*n* = 6–9 mice per group), cortical-to-medullary area ratio in thymic sections (**E**), and white pulp area of spleens (**F**) (*n* = 3–9 mice per group); * *p* < 0.05, *** *p* < 0.001, **** *p* < 0.0001 vs. UT-SMNΔ7. Data in graphs are expressed as mean ± SEM. Statistical analysis was performed using one-way ANOVA followed by Tukey’s post hoc test. (**G**–**N**) Representative H&E-stained paraffin sections of thymus and spleen from P14 mice in each experimental group. Blue and green arrows indicate the thymic cortex and medulla, respectively; dotted lines delineate the splenic white pulp. Scale bar in G = 100 µm (applies to **H**–**N**).

The pathological alterations in the thymus and spleen were examined in SMNΔ7 mice. Macroscopic morphology, size, and both absolute and relative weight, normalized to body weight, of organs were assessed in control and SMNΔ7 animals. At the end stage of the disease (P14), untreated SMNΔ7 mice exhibited a significant reduction in size and relative weights of the thymus and spleen compared to control littermates (Figure 2A–D).

Hematoxylin and eosin (H&E)-stained sections of thymus and spleen from control and SMNΔ7 mice were analyzed to assess SMA-induced structural alterations. The thymus, a primary lymphoid organ for T-cell development, is composed of encapsulated lobules separated by connective tissue septa. Each lobule contains a dense cellular cortex, rich in immature T cells, macrophages, and lymphoblasts, and a lighter medulla, mainly composed of mature T cells, epithelial clusters (Hassall’s corpuscles), and B cells. The corticomedullary junction harbors blood vessels and a mixture of T cells, dendritic cells, and B cells [30]. The spleen, a secondary lymphoid organ, consists of red pulp, containing connective tissue and vascular sinusoids, and white pulp surrounding central arterioles. The white pulp is subdivided into the periarteriolar lymphoid sheath (PALS), follicles, and marginal zone, with T cells predominantly in the PALS, B cells concentrated in the follicles, and macrophages and innate-like B cells in the marginal zone; macrophages are also distributed throughout both red and white pulp [31,32,33].

Compared with P14 control mice, untreated SMNΔ7 littermates displayed pronounced thymic abnormalities, including severe cortical depletion, reduced cortex-to-medulla ratio (Figure 2E,G,H), and enlargement of the medullary region. The spleens of untreated SMN∆7 mice also showed profound structural defects, characterized by markedly reduced cellular density in both red and white pulp, with the red pulp being most affected (Figure 2F,K,L). Overall, these findings are consistent with previous reports [16,26,27,28]. Importantly, SMN-ASO treatment partially or fully prevented thymic and splenic atrophy and histopathological changes, independently of the administration route (ICV or SC) (Figure 2C–F,I,J,M,N).

To assess whether the structural alterations detected at end-stage SMA (P14) were already present during early symptomatic stages, thymus and spleen samples from P7 control and SMNΔ7 mice were examined. At this age, no apparent macroscopic changes were observed when comparing organs from SMNΔ7 and control mice. Moreover, no significant differences were found in the relative weight of thymuses and spleens between SMNΔ7 and control animals (Supplementary Figure S1A,B). Histologically, the thymus of control mice displayed a normal cortical–medullary organization comparable to that observed at P14, and no overt abnormalities were detected in SMNΔ7 littermates (Supplementary Figure S1C,D). In contrast, the spleen of P7 control mice was still undergoing postnatal maturation and lacked fully defined white and red pulp domains (Supplementary Figure S1E,F). Therefore, it remains uncertain whether the splenic histopathology observed at P14 in SMNΔ7 mice is already initiated at P7 or rather emerges later as tissue organization becomes established.

### 2.4. SMA Thymus Exhibits T-Cell Maturation Defects, Which Are Prevented by SMN-ASO Treatments

**Figure 3:**
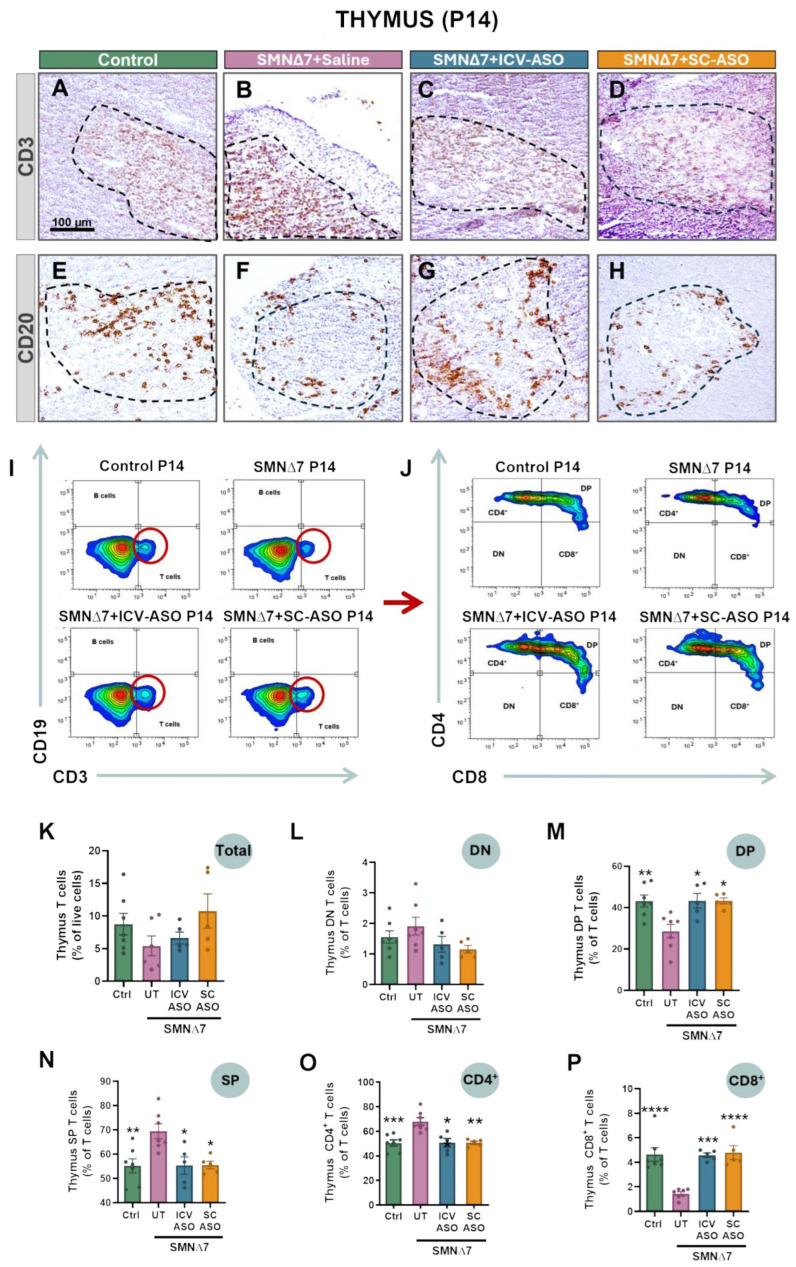
The thymus of untreated SMNΔ7 mice shows altered B- and T-cell distribution and abnormal T-cell development, both prevented by ICV or SC SMN-ASO treatment. (**A**–**H**) Representative CD20- and CD3-immunostained paraffin sections of thymus from P14 Ctrl and SMN∆7 mice, either untreated (UT) or treated with SMN-ASO via ICV or SC injections, as indicated. The thymic medulla is outlined with a dashed line (**A**–**H**). Scale bar in A = 100 µm (applies to all panels). (**I**) Representative fluorescence-activated cell sorting (FACS) plots of total thymocytes from P14 mice in the four experimental groups: control (Ctrl), untreated (UT) SMN∆7, ICV-ASO-treated SMN∆7, and SC-ASO-treated SMN∆7 mice. Thymocytes were surface-stained with anti-CD3 (T cells) and anti-CD19 (B cells). (**J**) CD3^+^ T cells (red gate in I) were further analyzed for CD4 and CD8 expression to identify four T-cell subsets: double negative (DN, CD4^−^CD8^−^), double positive (DP, CD4^+^CD8^+^), CD4 single positive (SP, CD4^+^CD8^−^), and CD8 SP (CD8^+^CD4^−^). (**K**) Percentage of CD3^+^ T cells relative to total live thymocytes. (**L**–**P**) Percentages of individual T-cell subsets (DN, DP, CD4^+^, CD8^+^) and combined single positive (SP = CD4^+^ plus CD8^+^) relative to total CD3^+^ T cells, as indicated (*n* = 5–7 mice per group). Data are presented as mean ± SEM. Statistical analysis was performed using one-way ANOVA followed by Tukey’s post hoc test: Black asterisks indicate significant differences between untreated SMN∆7 mice and Ctrl, SMN∆7+ICV-ASO, or SMN∆7+SC-ASO groups (* *p* < 0.05; ** *p* < 0.01; *** *p* < 0.001, **** *p* < 0.0001).

Given the key role of the thymus in T-cell maturation, the structural alterations observed in SMNΔ7 mice could reflect defects in T-cell development. T-cell precursors originating from hematopoietic stem cells migrate from the bone marrow to the thymic cortex as double-negative (DN, CD4^−^CD8^−^) cells, progress to double-positive (DP, CD4^+^CD8^+^), and subsequently differentiate into single-positive (SP, CD4^+^ or CD8^+^) T cells within the medulla before exiting to peripheral organs such as the spleen [30,34]. To investigate potential changes in thymic immune populations in P14 SMNΔ7 mice, we performed immunohistochemistry for CD3 and CD20 to detect T and B cells, respectively. In control thymus, CD3^+^ T cells were barely distinguishable in the densely packed cortex but were clearly visible in the less cellular medulla (Figure 3A), whereas CD20^+^ B cells were sparse and mainly restricted to the medulla, with only occasional cells in the cortex (Figure 3E). In the thymus of untreated SMNΔ7 mice, no apparent changes were noticed in CD3^+^ cell localization (Figure 3B). In contrast, CD20^+^ B cells exhibited an atypical redistribution, with cells appearing more widely scattered and frequently present outside the medulla (Figure 3F). Importantly, SMN-ASO treatment prevented these alterations, irrespective of administration route (Figure 3C,D,G,H).

To further characterize and quantify immune cell alterations in the SMNΔ7 thymus at P14, thymocytes were analyzed by flow cytometry. T and B cells were identified by CD3 and CD19 expression, respectively. Within the T-cell population, developmental stages were defined as DN (CD4^−^CD8^−^), DP (CD4^+^CD8^+^), and SP (CD4^+^CD8^−^ or CD4^−^CD8^+^), with SP cells further subdivided into helper (Th, CD4^+^CD8^−^) and cytotoxic (Tc, CD4^−^CD8^+^) subsets (Figure 3I,J). In untreated SMNΔ7 mice, total CD3^+^ T-cell numbers were slightly reduced compared to controls, although not significantly (Figure 3K). However, SMNΔ7 thymus displayed a modest increase in DN cells and a reduction in DP T cells (Figure 3L,M). In addition, a significant increase in the proportion of mature SP T cells was detected, driven by a higher amount of CD4^+^CD8^−^ Th cells and a concomitant reduction in the number of CD4^−^CD8^+^ Tc cells (Figure 3N–P). B cells were not detected by flow cytometry due to their low abundance in total thymic preparations. All these alterations in the SMN∆7 thymus were fully prevented by SMN-ASO treatment, irrespective of the administration route (ICV or SC) (Figure 3K–P). Flow cytometry at P7 revealed similar trends in T-cell developmental defects in SMNΔ7 mice; however, except for CD8^+^ cells, the differences in the proportions of individual T cell subpopulations did not reach statistical significance (Supplementary Figure S1G–N).

### 2.5. SMN-ASO Treatments Prevent Immune Cell Mislocation and Imbalance in the Spleen of SMNΔ7 Mice

**Figure 4:**
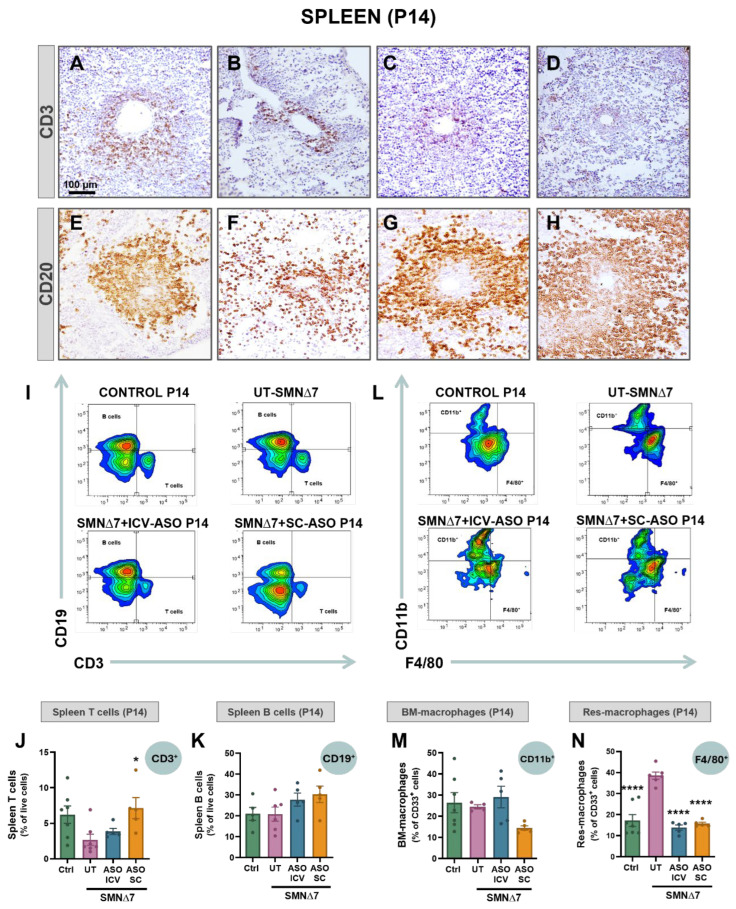
The spleen of untreated SMNΔ7 mice shows altered B- and T-cell distribution and immune cell population proportions, which are prevented by ICV or SC SMN-ASO treatment. (**A**–**H**) Representative CD3- and CD20-immunostained paraffin sections of spleens from P14 Ctrl and SMN∆7 mice, either untreated (UT) or treated with SMN-ASO via ICV or SC injections. Scale bar in (**A**) = 100 µm (applies to all panels). (**I**) Representative FACS plots of total splenocytes from P14 mice in the four experimental groups: control (Ctrl), untreated (UT) SMN∆7 mice, and ICV-ASO-treated and SC-ASO-treated SMN∆7 mice. Splenocytes were surface-stained with anti-CD3 (T cells) and anti-CD19 (B cells). (**J**,**K**) Percentages of T cells (CD3^+^) and B cells (CD19^+^), and relative to total live splenocytes, as indicated. (**L**) Myeloid cells expressing CD33 were further characterized using CD11b (bone marrow [BN]–derived macrophages) and F4/80 (spleen-resident [Res] macrophages) surface markers. (**M**,**N**) Percentages of CD11b^+^ and F4/80^+^ macrophage subsets relative to total CD33^+^ cells. Data are presented as mean ± SEM; *n* = 4–7 mice per group. Statistical analysis was performed using one-way ANOVA followed by Tukey’s post hoc test: Asterisks indicate significant differences between untreated SMN∆7 mice and Ctrl, SMN∆7+ICV-ASO, or SMN∆7+SC-ASO groups (* *p* < 0.05, **** *p* < 0.0001).

Immunohistochemical analysis of spleens from P14 mice was performed to evaluate lymphocyte density and spatial organization. In control spleens, CD3^+^ T cells were concentrated within the PALS, delineating a well-defined white pulp (Figure 4A,B). In contrast, SMNΔ7 spleens showed a markedly reduced density of CD3^+^ T cells in the PALS, with T cells scattered throughout the red pulp, consistent with an apparent decrease in their numbers. Similarly, CD20^+^ B cells, which in control spleens were predominantly localized around the PALS to form organized follicles (Figure 4E), displayed a diffuse staining pattern in SMNΔ7 spleens, with B cells dispersed throughout the red pulp and loss of organized PALS and white pulp structures (Figure 4F). Notably, both T- and B-cell abnormalities observed in SMNΔ7 spleens were fully prevented by SMN-ASO treatment, regardless of the administration route (ICV or SC) (Figure 4C,D,G,H).

Flow cytometry was used to further analyze the immune cell composition of spleens from control and SMNΔ7 mice, with or without SMN-ASO treatment. T cells (CD3^+^) and B cells (CD19^+^) were examined in detail (Figure 4I–K). At P7, no significant differences were observed between control and SMNΔ7 spleens in the proportions of T cells or B-cell subsets (Supplementary Figure S1M,N). By contrast, at P14, untreated SMNΔ7 mice displayed alterations in lymphocyte populations, characterized by a reduction in total T-cell numbers compared with controls (Figure 4J), whereas the proportion of B cells remained unchanged (Figure 4K). These results indicate that the disrupted B-cell organization observed by immunohistochemistry in the spleens of untreated SMN∆7 mice does not reflect a loss of B cells, but rather a defect in their maturation, which normally occurs in the bone marrow.

We next examined whether SMA affects the myeloid compartment, focusing on splenic macrophages. Two macrophage populations can be distinguished in the spleen based on their origin: hematopoietic stem cell (HSC)-derived macrophages, identified by CD11b expression, and resident macrophages of embryonic yolk sac origin, characterized by F4/80 expression [31,32,33]. Immunohistochemical analysis of F4/80 revealed a moderate increase in resident macrophages in untreated P14 SMNΔ7 spleens compared with controls, accompanied by a pronounced disruption of their normal localization in the marginal zone (Supplementary Figure S2A–E).

Flow cytometry analysis of CD11b^+^ and F4/80^+^ cells within the CD33^+^ myeloid compartment confirmed these observations. The spleens of untreated SMN∆7 mice exhibited a significant expansion of resident F4/80^+^ macrophages compared with controls, while the proportion of CD11b^+^ HSC-derived macrophages remained unchanged (Figure 4L–N). Importantly, both the increase in resident macrophages and the disruption of their spatial organization in SMA were fully prevented by SMN-ASO treatment, irrespective of the administration route (Figure 4L–N).

### 2.6. Bone Marrow B-Cell Development Is Impaired in SMA and Prevented by SMN-ASO Treatment

**Figure 5:**
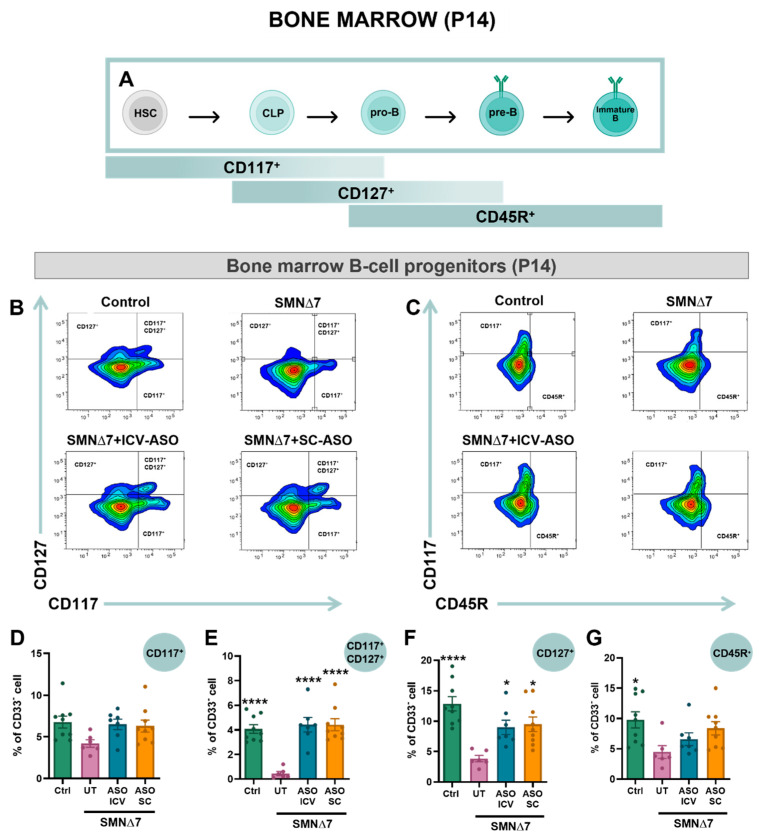
SMN∆7 mice exhibited impaired bone marrow B-cell maturation that is prevented by ICV or SC administration of SMN-ASO. (**A**) Schematic representation of the B-cell maturation process in the bone marrow, starting from pluripotent hematopoietic stem cells (HSCs), which give rise to common lymphoid progenitors (CLPs). CLPs progress through the pro-B and pre-B cell stages (the latter expressing membrane-bound IgM) and subsequently differentiate into immature B lymphocytes that migrate to secondary lymphoid organs. Each stage is characterized by the expression of specific surface markers (indicated in the scheme), which were used to distinguish the different populations by flow cytometry. (**B**) Representative FACS plots of CD33^−^ bone marrow cells from control (Ctrl), untreated (UT) SMN∆7, ICV-ASO–treated, and SC-ASO–treated SMN∆7 mice at P14. Four gates were defined based on CD117 and CD127 expression, identifying three B-cell progenitor populations: CD117^+^CD127^−^, CD117^−^CD127^+^, and CD117^+^CD127^+^. (**C**) Representative FACS plots of CD33^−^ bone marrow cells from the same four experimental groups, gated by CD117 and CD45R expression to identify two progenitor populations: CD117^+^ and CD45R^+^. (**D**–**G**) Quantification of B-cell progenitor subsets as a percentage of total CD33^−^ cells: CD117^+^ (**D**); CD117^+^CD127^+^ (**E**); CD127^+^ (**F**); CD45R^+^ (**G**). Data are presented as mean ± SEM (*n* = 6–9 mice per group). Statistical analysis was performed using one-way ANOVA followed by Tukey’s post hoc test. Black asterisks indicate significant differences between untreated SMN∆7 mice and Ctrl, SMN∆7+ICV-ASO, or SMN∆7+SC-ASO (* *p* < 0.05; **** *p* < 0.0001).

The structural alterations observed in the spleen of SMNΔ7 mice, together with the absence of changes in overall splenic B-cell proportions by flow cytometry, suggested potential defects in B-cell progenitors and maturation. Since B-cell development occurs in the bone marrow, a primary lymphoid organ not previously examined in SMA, we performed flow cytometry analysis of bone marrow cells from control and SMNΔ7 mice. Using antibodies against CD117, CD127, and CD45R, successive stages of B-cell development were distinguished (Figure 5A–C). Quantification of these populations (CD117^+^; CD117^+^CD127^+^; CD127^+^; CD45R^+^) revealed a reduction across different maturation stages in untreated SMNΔ7 mice compared to controls. Interestingly, this reduction was more pronounced in the most mature stages (i.e., CD117^+^CD127^+^; CD127^+^; CD45R^+^cells), indicating impaired B-cell maturation (Figure 5D–G). Importantly, SMN-ASO treatment preserved normal proportions of SMNΔ7 bone marrow B-cell subsets, with no significant differences between ICV and SC administration routes (Figure 5D–G).

### 2.7. Apoptosis Does Not Appear to Drive Cellular Depletion in Lymphoid Organs of SMN∆7 Mice

We next investigated whether the cellular depletion observed in the lymphoid organs of SMNΔ7 mice could result from increased apoptotic cell death. Flow cytometry analysis was performed using Annexin V-APC (AnxV) as a marker of apoptosis, together with 7-aminoactinomycin D (7-AAD) to assess cell viability. Cells positive for AnxV alone were considered to be in early-stage apoptosis, whereas double-positive cells (AnxV^+^7-AAD^+^) were indicative of late-stage apoptosis or secondary necrosis [35,36]. No significant differences were detected in the proportions of B or T lymphocytes undergoing early or late apoptosis in the thymus or spleen across the four experimental groups (Supplementary Figure S3A–L). These results indicate that increased apoptosis is not the primary driver of lymphoid cellular depletion in SMNΔ7 mice.

### 2.8. Thymic, but Not Splenic, Vascular Density Is Altered in SMN∆7 Mice

Altered vascular supply could contribute to lymphoid architectural defects and immune abnormalities in SMA. To assess this, immunofluorescence for CD31, an endothelial cell marker, was performed in control and SMN∆7 thymuses and spleens. Consistent with previous reports [26,27], splenic vascular density was comparable between SMNΔ7 and control mice (Supplementary Figure S4A–K), suggesting the splenic alterations observed at late stages of SMA are unlikely due to vascular defects. In contrast, the SMNΔ7 thymus displayed region-specific vascular abnormalities: cortical vessel density was normal, but CD31^+^ vessels were significantly reduced in the medulla. These findings suggest that impaired thymic medullary vascularization may contribute to the defective immune phenotype in SMA. Defects in thymic vascularization were partially rescued by SMN-ASO treatment, regardless of administration route (ICV or SC) (Supplementary Figure S4A–K).

### 2.9. SMN-ASO Treatment Prevents IL-6–Mediated Inflammation Induced by SMN Deficiency in Lymphoid Organs

**Figure 6:**
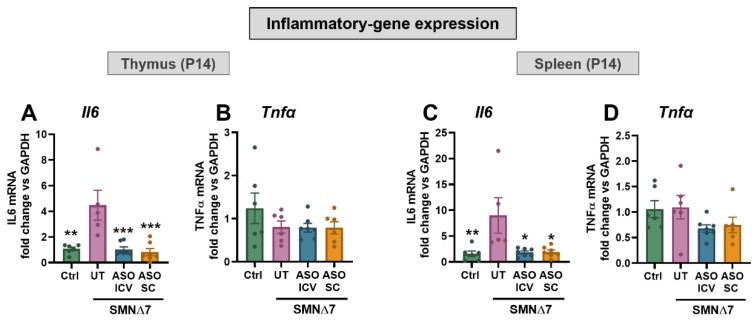
Altered expression of pro-inflammatory cytokines *Il6* and *Tnfα* in the thymus and spleen of SMN∆7 mice is prevented by ICV or SC SMN-ASO treatment. (**A**–**D**) Relative mRNA levels of *Il6* (**A**,**C**) and *Tnfα* (**B**,**D**) in thymus (**A**,**B**) and spleen (**C**,**D**) of P14 control (Ctrl), untreated (UT) SMN∆7, ICV-ASO–treated, and SC-ASO–treated mice. Data are presented as mean ± SEM and normalized to *Gapdh* mRNA expression; *n* = 5–7 mice per group. Statistical significance was determined by one-way ANOVA followed by Tukey’s post hoc test. Asterisks indicate significant differences between untreated SMN∆7 and Ctrl, SMN∆7+ICV-ASO, or SMN∆7+SC-ASO groups (* *p* < 0.05, ** *p* < 0.01, *** *p* < 0.001).

To assess whether SMN deficiency induces inflammation in lymphoid tissues, we measured tumor necrosis factor-α (TNF-α) and interleukin-6 (IL-6) expression by RT-qPCR in thymus and spleen (Figure 6A–D). TNF-α levels were unchanged across all groups, indicating that SMN deficiency and ASO treatment do not impact TNF-α transcription in these tissues. In contrast, IL-6 was significantly elevated in the thymus and spleen of untreated SMNΔ7 mice, reflecting a local pro-inflammatory state associated with SMN deficiency. Remarkably, both ICV and SC SMN-ASO treatments fully prevented IL-6 upregulation, restoring expression to control levels (Figure 6A,C). These results indicate that, beyond correcting splicing defects, SMN-ASO treatment also counteracts the aberrant inflammatory response in peripheral lymphoid organs associated with SMN deficiency.

### 2.10. Thymic and Splenic Pathology in SMA Fetuses and Patients with One or Two SMN2 Copies Predicted to Develop Severe Disease

To assess whether similar thymic and splenic abnormalities occur in human SMA, autopsy samples from severe SMA fetuses and patients and age-matched controls were analyzed at different developmental stages (Supplementary Figure S5A).

**Figure 7:**
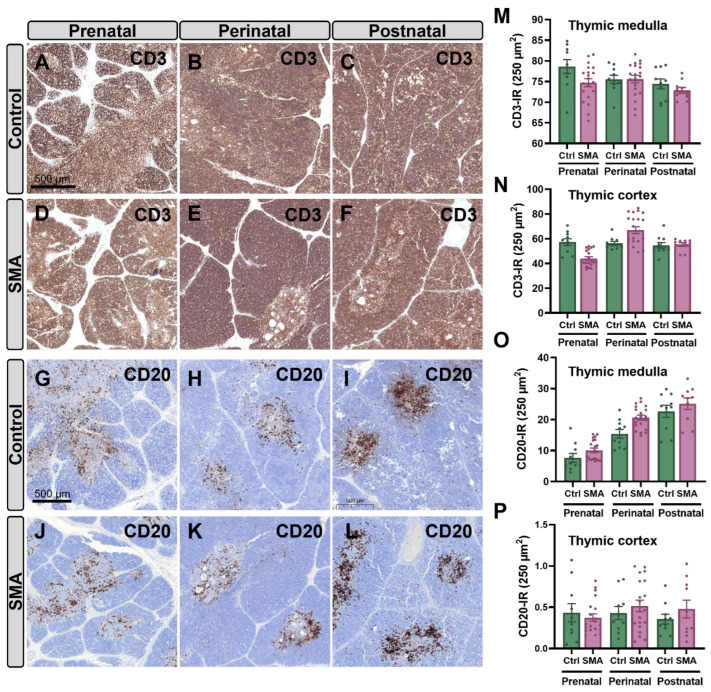
Immunohistochemical analysis of CD3 and CD20 in the thymus of control and SMA prenatal, perinatal, and postnatal human samples. (**A**–**L**) Representative images of CD3 (**A**–**F**) and CD20 (**G**–**L**) immunostaining in prenatal, perinatal, and postnatal thymic sections, as indicated. Scale bar = 500 μm in (**A**,**G**) (applies to (**B**–**F**) and (**H**–**L**), respectively). (**M**–**P**) Quantification of CD3- and CD20-immunoreactive areas in the thymic cortex and medulla, as indicated. Data are presented as mean ± SEM. No significant differences were observed between groups at any developmental stage (one-way ANOVA followed by Tukey’s post hoc test). Sample sizes are provided in Supplementary Figure S5A.

**Figure 8:**
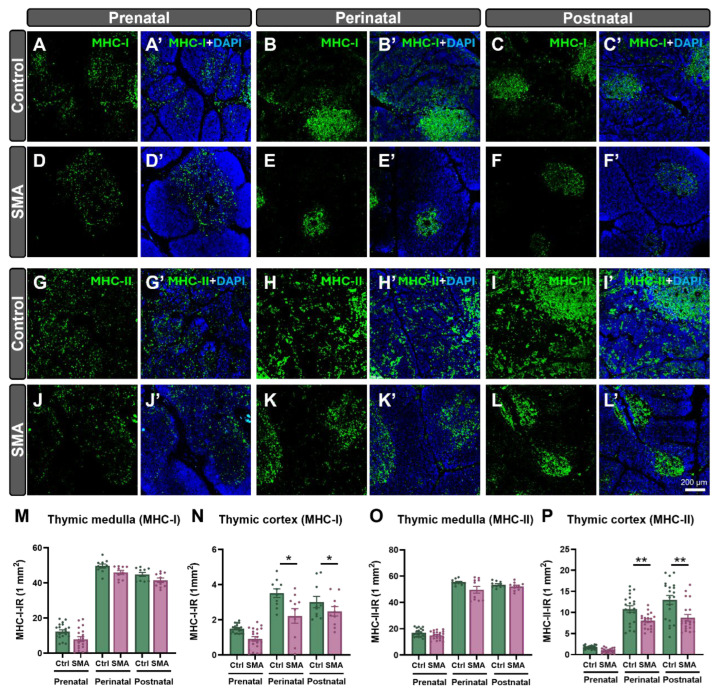
The thymus of SMA fetuses and patients exhibits a decreased expression of MHC-I and MHC-II molecules. (**A**–**L’**) Immunofluorescence staining for MHC-I (**A**–**F’**, green) and MHC-II (**G**–**L’**, green) in sections of thymus from control and SMA samples at prenatal, perinatal stages, and postnatal stages, as indicated. Nuclei were counterstained with DAPI (blue). Scale bar in **L’** = 200 µm (applies to **A**–**L**). (**M**–**P**) Quantification of MHC-I^+^ (**M**,**N**) and MHC-II^+^ (**O**,**P**) cell density in the thymic medulla (**M**,**O**) and cortex (**N**,**P**) of control and SMA patients at the different developmental stages examined. Positive cell density for MHC-I and MHC-II was determined in areas of 1 mm^2^. Data are shown as mean ± SEM. * *p* < 0.05, ** *p* < 0.01 (unpaired *t*-test). Sample sizes are provided in Supplementary Figure S5A.

In contrast to findings in the SMN∆7 mouse model, no significant alterations in thymic histoarchitecture were detected in human SMA samples at any developmental stage compared to controls (Supplementary Figure S5B–H). Immunohistochemical analysis using anti-CD3 and anti-CD20 antibodies, which label T and B lymphocytes, respectively, revealed no significant differences in the density of these cell populations between SMA and control samples of thymus at prenatal, perinatal, and postnatal stages (Figure 7A–P). To further investigate potential immune-related alterations in the thymus associated with SMA pathology, we assessed the expression of MHC-I and MHC-II molecules, which are key antigen-presenting proteins that enable immune recognition by CD8^+^ (cytotoxic) and CD4^+^ (helper) T cells, respectively [37] (Figure 8A–P). In both control and SMA thymic tissue, major histocompatibility complex class I (MHC-I) and class II (MHC-II) immunoreactivity was mainly localized to the medulla, with scattered positive cells in the cortex, and both markers increased progressively with development. However, during the perinatal stage, SMA thymuses displayed a significant reduction in the density of MHC-I– and MHC-II–positive cells in the cortex (~37% and ~25% decrease, respectively), which persisted postnatally (~17% reduction in MHC-I and ~32% reduction in MHC-II relative to controls) (Figure 8N,P).

**Figure 9:**
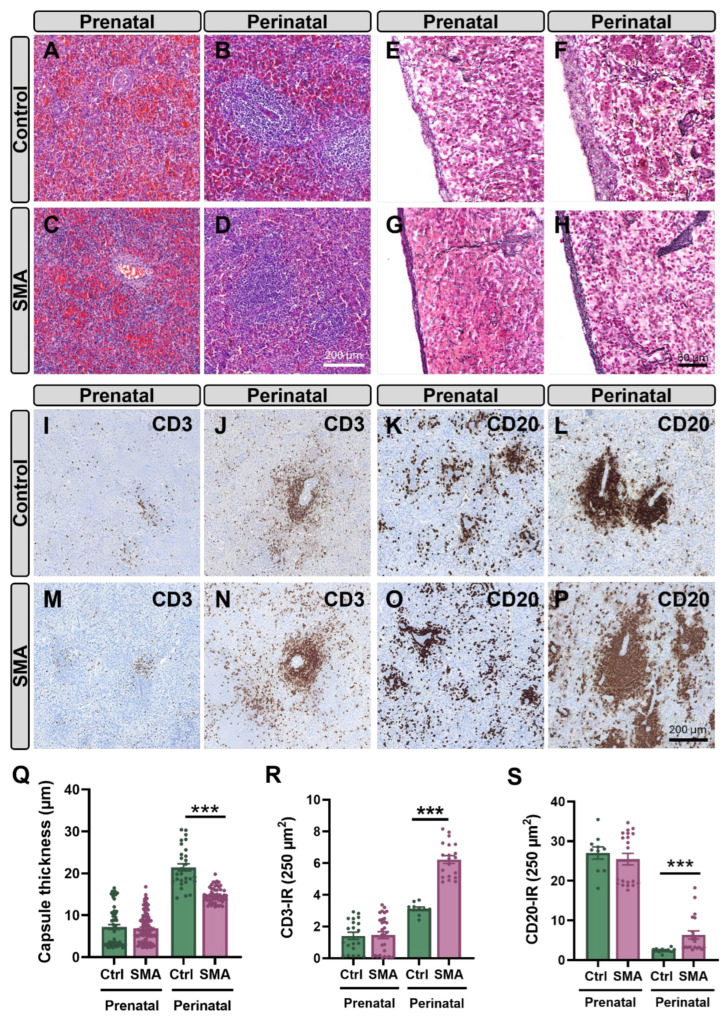
Histopathological changes in human SMA prenatal and perinatal spleens. (**A**–**H**) Representative images of H&E—(**A**–**D**), and reticulin—(**E**–**H**) stained sections from human spleens of control and SMA fetuses and patients at prenatal and perinatal stages. (**I**–**P**) Representative images of human spleen sections from the different experimental groups immunoperoxidase-stained with antibodies anti-CD3 (**I**–**N**) and CD20 (**K**–**P**), as indicated. Scale bars = **D**, 200 µm (applies to **A**–**C**); H, 50 µm (applies to **E**–**G**); (**P**) 200 μm (applies to **I**–**O**). (**Q**–**S**) Quantification of spleen capsule thickness (**Q**), and the density of CD3+ and CD20+ cells in the splenic red pulp. IR = immunoreactivity. The density of cells immunopositive for CD3 and CD20 was calculated in areas of 250 µm^2^. Data are presented as mean ± SEM; *** *p* < 0.001 (unpaired *t*-test); sample sizes are provided in Supplementary Figure S5A.

In contrast to the relatively preserved thymus, the spleen of human SMA samples showed marked structural abnormalities compared to age-matched controls. At the prenatal stage, the distinction between red and white pulp compartments was still developing and, therefore, appeared less defined in both control and SMA spleens. At perinatal stages, however, SMA spleens’ white pulp boundaries were particularly poorly demarcated, and the red pulp volume appeared to be further reduced compared to controls (Figure 9A–D). Moreover, reticulin fiber staining revealed a significant reduction (~30%) in the capsule thickness and fiber density within the splenic capsule of SMA samples compared to controls (Figure 9E–H,Q). Immunohistochemistry for CD3 and CD20 at the prenatal stage showed low expression of both antigens in SMA and control spleens, with no significant differences between groups (Figure 9I,M,K,O,R,S). By the perinatal stage, control spleens displayed clearly organized PALS, with CD3^+^ T cells surrounding central arterioles and CD20^+^ B cells located in adjacent regions. In SMA spleens, however, the red pulp exhibited a marked increase in CD3 (~151%) (Figure 9I–N,R) and CD20 (~247%) (Figure 9K–P,S) immunoreactivity compared to controls.

**Figure 10:**
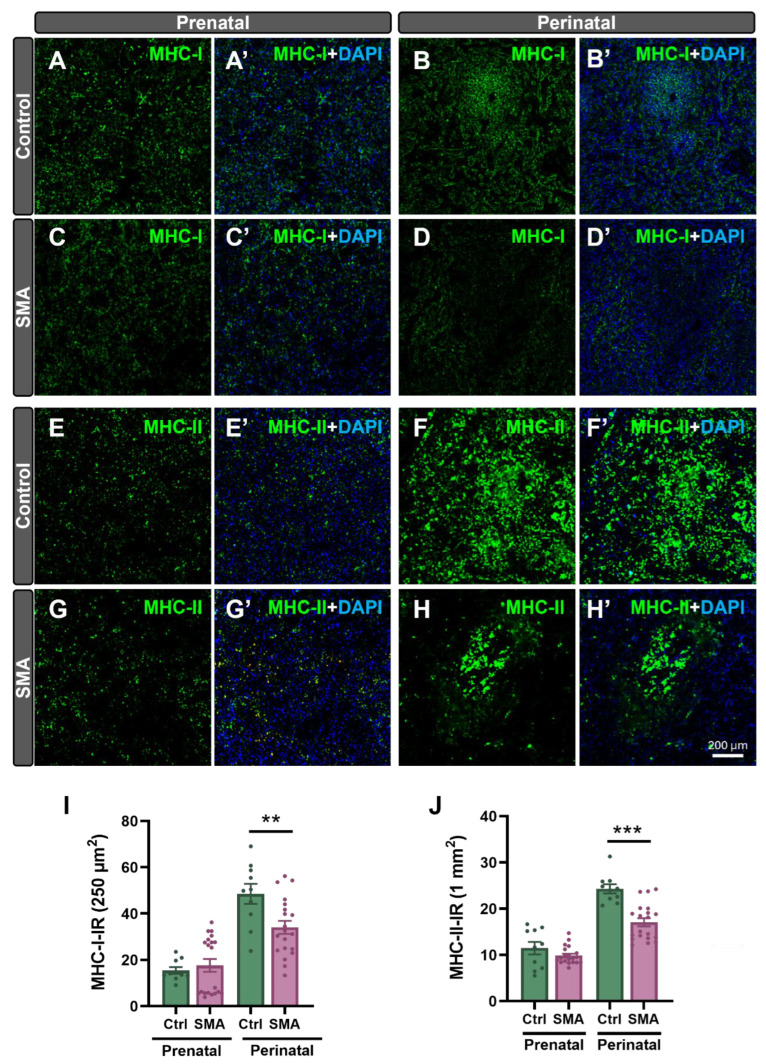
Decreased MHC-I and MHC-II expression in human SMA prenatal and perinatal spleens (**A**–**H’**) Immunofluorescence staining for MHC-I (**A**–**D’**, green) and MHC-II (**E**–**H’**, green) in spleen sections from control and SMA patients at prenatal and perinatal stages, as indicated. Nuclei were counterstained with DAPI (blue). Scale bar in **H’** = 200 µm (applies to **A**–**H**). (**I**,**J**) Quantification of MHC-I^+^ (**I**) and MHC-II^+^ (**J**) cell density in control and SMA spleens at prenatal and perinatal stages. Positive cell density for MHC-I and MHC-II was calculated in areas of 250 µm^2^ and 1 mm^2^, respectively. Data are shown as mean ± SEM. ** *p* < 0.01, *** *p* < 0.001 (unpaired *t*-test). Sample sizes are provided in Supplementary Figure S5A.

MHC immunostaining further highlighted defects in splenic compartmentalization. While MHC-II immunoreactivity was generally stronger than MHC-I across both groups and stages, SMA spleens showed altered patterns. In controls, MHC-I was strongly expressed around arterioles, whereas in SMA samples, its distribution was diffuse, suggesting impaired white pulp organization (Figure 10A–D’). At the perinatal stage, MHC-II remained prominent around arterioles in both groups but was reduced in the red pulp of SMA spleens (Figure 10E–H’). Quantification revealed significant decreases in both MHC-I (~28%) and MHC-II (~32%) immunoreactivity in perinatal SMA spleens compared to controls (Figure 10I,J).

## 3. Discussion

SMA has long been defined as an MN disease, but growing evidence indicates that reduced levels of SMN protein affect several tissues and systems beyond the neuromuscular axis [21,23,24]. In line with our previous reports [16] and others [16,26,27,28], the current study further characterized the impact of SMN deficiency on the immune system, showing that severe SMA is associated with profound structural and cellular alterations in both primary (thymus and bone marrow) and secondary (spleen) lymphoid organs in the SMNΔ7 mouse. These alterations include marked thymic cortical atrophy, disorganization of splenic white pulp architecture, abnormal lymphocyte subset composition, impaired B-cell maturation in the bone marrow, and expansion of splenic resident macrophages. Consistent with these findings, postmortem analysis of lymphoid organs from SMA fetuses and patients revealed analogous splenic alterations and subtle thymic changes, suggesting that similar immune-related abnormalities may also occur in humans. Functionally, these defects are likely to compromise immune competence in SMA. Together, these results point to an intrinsic SMN-dependent requirement for proper immune organ development and lymphocyte maturation. Importantly, these defects were largely prevented by early SMN restoration therapy, supporting a direct and developmentally critical role for SMN in immune system integrity.

### 3.1. Potential Mechanisms Underlying Lymphoid Tissue Abnormalities and Disease Progression in SMA

The thymus is essential for T-cell development and selection, providing a specialized microenvironment for thymocyte differentiation, T-cell receptor gene rearrangement, and both positive and negative selection of developing T-cells [38,39]. The spleen, in turn, plays key roles in immune surveillance, the filtering of blood-borne antigens, and the activation and maturation of B cells within well-organized white pulp structures [40]. Disruption of lymphoid organ architecture and cell composition, such as altered CD4^+^/CD8^+^ T-cell balance or B-cell mislocalization, could impair the adaptive immune response. Furthermore, the observed reduction in early B-cell precursors in bone marrow suggests a defect in hematopoiesis that may underlie the splenic phenotype observed in SMA [41]. These abnormalities may contribute to disease progression by disrupting neuroimmune crosstalk, impairing tissue repair, or fostering chronic inflammation. Indeed, studies in SMA models and patients suggest that neuroinflammation and immune dysfunction contribute significantly to the pathogenesis and severity of the disease [28,29,42].

Importantly, cellular depletion in lymphoid organs in SMN∆7 mice does not appear to result from increased apoptosis, as AnxV/7-AAD assays revealed no significant differences relative to control animals. However, in our previous study using a more severe SMA mouse model [16], we observed increased cell death in the thymus, suggesting a threshold for SMN levels, below which lymphocyte loss may occur. Consistent with this, analysis of the intermediate SMA mouse model *Smn^2B/−^* [28] has shown no transcriptional upregulation of pro-apoptotic genes in the thymus. However, increased TUNEL labeling and caspase-3 activation were reported at late stages of the disease, pointing to potential post-transcriptional regulation or context-specific apoptosis. It is plausible that the cellular depletion observed in lymphoid organs of SMNΔ7 mice arises not only from subtle or non-canonical forms of cell death (e.g., necroptosis, pyroptosis, or autophagy-associated death) that escape detection by AnxV-based assays [43,44], but also from intrinsic defects in lymphocyte development. In support of this, our current study revealed a marked reduction in early B-cell precursors in the bone marrow of SMNΔ7 mice. Additionally, immunohistochemical analysis demonstrated notable alterations in CD20^+^ B-cell distribution within the SMNΔ7 splenic white pulp, indicating defective maturation and/or mislocalization of B cells. Future studies combining lineage tracing with markers of alternative cell death pathways will be essential to clarify these mechanisms and define the precise contribution of hematopoietic dysfunction to SMA-associated immune defects.

In addition to developmental and non-apoptotic cell loss mechanisms, recent studies have highlighted the role of SMN in proteostasis maintenance, through its chaperoning role in RNP assembly, with links to major protein-folding systems such as Hsc70/HspA family members [45]. Disruption of this chaperone function may result in the accumulation of misfolded proteins and aberrant RNPs, which can, in turn, activate innate immune pathways. This emerging connection between SMN deficiency, proteostasis imbalance, and innate immune activation may represent an additional contributor to the immune abnormalities observed in SMA, complementing the hematopoietic and maturation defects described above [46,47].

Remarkably, our histological examination of lymphoid organs in SMNΔ7 mice did not reveal overt vascular defects in the spleen. This aligns with Khairallah et al. [27], who also observed preserved splenic vasculature despite profound architectural disruption. However, in contrast to the spleen, we detected vascular alterations in the thymic medulla of SMNΔ7 mice, indicating that vascular involvement may occur in a tissue-specific manner. Together, these findings suggest that the structural and cellular abnormalities observed in lymphoid tissues are unlikely to result solely from vascular insufficiency but may reflect a combination of direct effects of SMN deficiency within hematopoietic or stromal compartments and localized vascular dysfunction. Microvascular alterations may also act as modulators of the broader SMA phenotype. Endothelial dysfunction and impaired angiogenesis have been linked to SMN deficiency [24,48,49,50], and lymphoid organs are highly dependent on intact microvascular networks to sustain tissue organization, immune cell trafficking, and metabolic support [51]. While early-stage pathology in severe models such as SMNΔ7 mice shows limited involvement of the splenic microvasculature, localized impairments in thymic perfusion could exacerbate structural disruption. Over longer disease courses, slower-progressing or treated SMA models frequently develop distal tissue necrosis in tails and ears, and microvascular degeneration, reflecting a late-onset vascular component of the disease [52,53]. Thus, we cannot exclude the possibility that broader vascular impairments may emerge over extended disease courses. Importantly, in milder SMA models resembling type III disease, such as those carrying four copies of SMN2, SMN-ASO treatment reduces but does not fully prevent the incidence of distal necrosis [52]. This partial rescue supports a protective, though incomplete, effect of SMN restoration on peripheral vascular integrity. In fact, we have observed comparable distal necrotic lesions and potential microvascular alterations in long-term SMN-ASO-rescued SMNΔ7 mice (unpublished observations). Collectively, these observations suggest that vascular complications may arise both as tissue-specific features in lymphoid organs, such as the thymus, and as late-stage manifestations of prolonged survival. Additionally, Kothary’s group [54] has reported reduced splenic blood flow in the *Smn^2B/−^* model, which has a longer lifespan than the SMNΔ7 mouse. This further supports the idea that vascular phenotypes in SMA are modulated by model severity, tissue specificity, and disease duration. Thus, while early-stage pathology in severe models like the SMNΔ7 mouse shows no overt vascular involvement in the spleen, but vascular alterations in the thymus, intermediate or chronic SMA models may develop secondary circulatory impairments that contribute to distal tissue pathology.

### 3.2. Human SMA Immune Pathology: Molecular and Structural Parallels with Mouse Models

While thymic and splenic alterations were prominent in the SMNΔ7 mouse model, only partial parallels were observed in type I SMA patients. In human samples, thymic architecture and immune cell composition appeared largely preserved across the examined developmental stages, contrasting with the severe cortical thymocyte depletion seen in mice. In contrast, the spleen of SMA patients displayed abnormalities more closely aligned with those observed in murine models, including reduced distinction between red and white pulp and aberrant clustering or increased density of immune cells in inappropriate regions.

The conservation of splenic abnormalities between SMA mice and human patients supports a biologically relevant role for immune involvement in the disease. In this regard, the histological analysis of perinatal human SMA spleens revealed novel aspects of pathology. Despite normal prenatal development, perinatal SMA spleens exhibited a reduction in the thickness of the capsule, a structure that allows for spleen expansion and rapid blood discharge during systemic hypoxia [32]. Although this finding has not been reported in other pathologies, its potential impact on splenic reserve function and oxygenation responses warrants further investigation. Moreover, the architecture of red and white pulp appeared disrupted in perinatal human SMA spleens, with reduced red blood cell content in the red pulp and increased infiltration of B- and T-cells in this compartment, possibly reflecting delayed or abnormal lymphocyte localization. Germinal centers were present but appeared immature compared to age-matched controls, suggesting delayed or defective immune compartmentalization. These findings closely parallel those reported in SMNΔ7 and *Smn^2B/−^* mice, where splenic pulp disorganization and B- and T-cell mislocalization have been described [27,28]. These similarities reinforce the notion that SMN deficiency directly impairs spleen ontogeny and immune maturation from early developmental stages.

In contrast to findings in SMA mouse models, the thymus of human SMA tissue appeared structurally preserved across the prenatal and early postnatal stages examined. Parameters such as the cortico-medullary ratio, overall cellularity, and the distribution of B- and T-cell populations did not show significant deviations from age-matched controls. This diverges from observations in mice, where cortical thinning and impaired T-cell development have been documented [28], and suggests that thymic pathology in human SMA may be delayed in disease progression, subtle, or subclinical.

Nonetheless, despite preserved architecture, perinatal human SMA lymphoid tissues revealed molecular alterations. Both MHC-I and MHC-II expression were reduced in the thymic cortex and spleen, particularly at perinatal stages, potentially compromising CD8^+^/CD4^+^ T-cell maturation and antigen presentation [55]. Since MHC-II is particularly crucial for thymocyte education via antigen presentation by B cells in the thymus, its downregulation may impair negative selection and central tolerance mechanisms [56,57]. Given that thymic B cells express high levels of MHC-II and contribute to the deletion of autoreactive T cells and regulatory T cell induction, their dysfunction may alter thymocyte differentiation [58]. Consequently, SMN deficiency could promote the generation of unconventional T-cell subsets, which have been linked to pediatric immune dysregulation syndromes [59].

Our data support the translational value of the SMNΔ7 mouse model for investigating peripheral immune involvement in SMA. At the same time, they underscore species-specific differences in the immune system organization, development, and pathology [60]. Such discrepancies must be carefully considered when extrapolating mouse findings to humans. Several factors may account for these divergences. First, the postnatal progression of type I SMA in humans is typically more aggressive than in SMA mouse models, potentially limiting the window during which immune alterations can manifest [5,61]. Consistent with this, clear pathology is readily detectable in neuromuscular structures (MN, axon, neuromuscular junction, and muscle) during human development, supporting the main involvement of the neuromuscular system in the manifestations of the disease [62]. Second, immune ontogeny differs markedly between species. In mice, hematopoietic and lymphoid development occurs rapidly in early postnatal life, with defined waves of progenitor emergence and tissue colonization [63]. In contrast, human immune maturation is more protracted and influenced by environmental exposures over an extended period [60,64]. Furthermore, factors such as perinatal care, respiratory support, and reduced infection burden in clinical settings may partially mask or delay the manifestation of immune abnormalities in human patients. Importantly, the notion that disease severity and lifespan shape the emergence of pathological features extends beyond immune organs and also applies to canonical hallmarks of SMA, such as MN loss. For example, previous studies by our group have shown that in severe SMA mouse models, such as the *Smn^−/−^;SMN2^+/+^* (Burghes’ severe model) and the SMNΔ7, only about 20% of lumbar spinal MN are lost by end-stage disease [15,16]. Despite marked motor deficits, the rapid progression of the disease in these models leads to death before extensive neurodegeneration can occur. In contrast, in the intermediate *Smn^2B/−^* model, which has an extended lifespan, MN loss can reach up to ~60% [65]. Thus, the apparent absence of pathology in certain tissues, including lymphoid organs, may reflect insufficient time for lesion development rather than true preservation, and may be underestimated in more severe or rapidly progressive models. As early therapeutic interventions improve survival [66,67], these peripheral manifestations may become more apparent, particularly in tissues that rely on sustained SMN expression for proper development. This emphasizes the need to monitor immune parameters in long-term treated SMA patients and to consider peripheral organs as important targets in future therapeutic strategies.

Taken together, our comparative analysis suggests that while structural thymic defects may be less evident in human SMA during early development, molecular and immunophenotypic changes, particularly in the spleen, do emerge perinatally and mirror findings in mouse models. These observations provide further support for the relevance of immune monitoring and therapeutic targeting of lymphoid organs in SMA patients, particularly those with prolonged survival enabled by current therapies.

### 3.3. Systemic and CNS-Directed SMN-ASO Delivery Prevents Immune and Inflammatory Defects in SMA

One of the most relevant outcomes of this study is the demonstration that early SMN restoration via ASO therapy effectively prevents immune organ abnormalities in SMNΔ7 mice. Both SC and ICV administration of SMN-ASO provide comparable rescue of lymphoid architecture and immune cell maturation, demonstrating that peripheral and CNS-directed delivery routes are similarly effective. Notably, our observation that SC administration alone can correct a peripheral immune pathology represents a novel and clinically relevant advance. To our knowledge, this is the first evidence that systemic pharmacological delivery of an SMN-enhancing agent can normalize immune organ structure and hematopoietic cell development in SMA mice. Previous studies have primarily focused on ICV delivery; for instance, Khairallah et al. [27] have demonstrated that ICV administration of SMN-ASO in SMNΔ7 mice rescues splenic architecture, which is consistent with our findings and reinforces the therapeutic potential of early SMN restoration. Likewise, Deguise et al. [28] have shown that genetically increasing SMN levels in *Smn^2B/−^* mice by introducing additional copies of the human *SMN2* gene also ameliorated lymphoid tissue defects, further supporting the concept that adequate SMN expression is directly required for immune organ development. However, unlike our pharmacological approach, this genetic strategy involves constitutive systemic overexpression, which, while informative, may not fully capture the translational applicability of drug-based treatments.

Interestingly, the beneficial effect of ASO ICV delivery on SMA lymphoid defects, despite being primarily CNS-directed, provides important mechanistic insight. In neonatal mice, the blood–brain barrier (BBB) is not fully mature and exhibits increased permeability, allowing ICV-administered ASOs to reach peripheral tissues [68,69,70]. This transient leakiness likely explains the observed rescue of immune phenotypes following early postnatal ICV treatment. However, as the BBB matures in later developmental stages and is already largely sealed in postnatal humans, drug passage from cerebrospinal fluid into circulation becomes limited [70]. Therefore, the peripheral efficacy seen with ICV administration in neonatal mouse models may not fully translate clinically, especially when treatment is initiated after birth. This highlights the therapeutic advantage of systemic approaches, such as SC ASO delivery, oral *SMN2* splicing modulators like risdiplam, or systemically delivered gene therapies, which bypass the BBB and ensure adequate SMN restoration in peripheral organs, including lymphoid tissues. The full prevention of immune abnormalities in SMA mice by SMN-ASO, regardless of delivery route, together with the supporting evidence from both genetic and pharmacological interventions, suggests that these defects are not merely secondary consequences of MN loss or disease severity but reflect a direct requirement for SMN in immune organ development.

Beyond correcting developmental defects in lymphoid organs, our findings uncover an additional role of SMN restoration in dampening local inflammation. Specifically, SMN deficiency was associated with a marked upregulation of *Il6* expression in both the thymus and spleen of untreated SMNΔ7 mice, indicating an IL–6–mediated pro-inflammatory response within these tissues. Notably, SMN-ASO treatment, via either ICV or systemic (SC) delivery, fully normalized *Il6* transcript levels in SMN∆7 mice, while Tnfα expression remained unchanged, suggesting a selective cytokine dysregulation in this model. These results expand on previous reports from other SMA models: while Kothary’s group has observed no major cytokine changes in the *Smn^−/−^;SMN2^+/+^* mouse, and the *Smn^2B/−^* model only exhibited cytokine induction in the thymus [28], our data reveal a broader inflammatory involvement in SMNΔ7 mice, affecting both primary and secondary lymphoid organs. This partially mirrors observations in SMA patients, where elevated circulating IL-6 and TNF-α have been detected, particularly in pediatric cohorts [71]. Collectively, these findings suggest that SMN restoration not only re-establishes lymphoid organ architecture and immune cell composition but also suppresses aberrant inflammatory signaling. The ability of SMN-ASO therapy to modulate local cytokine expression reinforces its systemic therapeutic benefits and highlights immune dysregulation as a treatable component of SMA pathology. Moreover, our findings underscore the importance of model selection when investigating immune aspects of SMA, as cytokine expression patterns appear to vary significantly across commonly used mouse models.

### 3.4. Future Directions and Limitations

Our findings reinforce the multisystemic nature of SMA and highlight the need for broader clinical evaluation beyond the neuromuscular axis. Although current SMN-restoring therapies such as nusinersen and onasemnogene abeparvovec have markedly improved survival and motor function [72,73], peripheral manifestations remain insufficiently addressed in both clinical monitoring and therapeutic design. Here, we demonstrate that early SMN-ASO treatment fully prevents structural and inflammatory abnormalities in lymphoid organs, irrespective of CNS-targeted or systemic delivery. This indicates that adequate SMN expression is also required in peripheral compartments, including the immune and hematopoietic systems. Taken together, our findings challenge treatment paradigms directed at the CNS alone and support the implementation of systemic therapeutic strategies that better reflect the full complexity of SMA pathophysiology [22,23,24]. Future therapeutic approaches may benefit from combining central and peripheral SMN delivery (e.g., intrathecal plus oral or intravenous administration) or employing optimized delivery platforms that ensure broad biodistribution [74,75]. Such strategies could mitigate not only neuromuscular deficits but also peripheral dysfunctions, including immune dysregulation, metabolic alterations, and organ-specific developmental defects, which are expected to gain clinical relevance as SMA patient lifespan increases [67]. Ultimately, expanding the therapeutic scope to include peripheral organs will be essential to improving long-term health and reducing secondary complications in SMA.

However, several limitations of our study should be acknowledged. First, we did not assess the functional capacity of the immune system to respond to physiological or infectious challenges, which limits conclusions regarding the clinical impact of the observed immune alterations. Second, although SMN-ASO treatment increased *SMN2* exon 7 inclusion in both central and peripheral tissues, it remains unclear whether immune phenotypic rescue is driven by local SMN restoration within lymphoid organs or from broader systemic effects. Nonetheless, our detection of elevated *SMN* mRNA levels in both the thymus and spleen following treatment supports the notion that direct restoration within lymphoid organs may contribute to the observed improvements. Finally, important species-specific differences in developmental timing, immune system maturation, and treatment protocols between mice and humans must be carefully considered when evaluating the translational relevance of these findings [60,76,77].

## 4. Methods

### 4.1. Animals and ASO Treatments

The SMNΔ7 mouse model, which recapitulates a severe form of SMA, was used in this study. Heterozygous *Smn^+/−^;SMN2^+/+^;SMNΔ7^+/+^* mice (FVB.Cg-*Grm7^Tg(SMN2)89Ahmb^ Smn1^tm1Msd^* Tg(SMN2*delta7)4299Ahmb/J, strain #005025) were purchased from The Jackson Laboratory (Sacramento, CA). Heterozygous male and female animals were crossed to obtain *Smn^−/−^;SMN2^+/+^;SMNΔ7^+/+^* (homozygous knockouts for the *Smn* gene, SMNΔ7) and *Smn^+/+^;SMN2^+/+^;SMNΔ7^+/+^* (homozygous for the murine *Smn* gene, hereafter referred to as control) mice. Offspring genotypes were determined by PCR-based genotyping of genomic DNA obtained from tail biopsies, as described below. SMN∆7 mice have a mean lifespan of 13.3 ± 0.3 days [78]. Since no significant sex-specific differences in disease phenotype or treatment response have been found in the SMNΔ7 mouse model ([79] and our unpublished data), both male and female animals were included in the study without distinction. Mice were housed in the University of Lleida Animal Care Facility under a 12 h light/dark cycle with ad libitum access to standard laboratory chow and water. All experimental procedures were approved by the Institutional Animal Care and Use Committee of the University of Lleida and conducted in accordance with the European Council Directive and the regulations established by the Generalitat de Catalunya.

On the day of birth (P0), the mice’s paws were marked with tattoo paste (Ketchum Manufacturing, Brockville, Canada) for identification purposes, and a small tail biopsy was collected for genotyping. Genomic DNA extraction and polymerase chain reaction (PCR) setup were performed using the PhireTissue Direct PCRMaster Mix commercial kit (Thermo Fisher Scientific, Waltham, MA, USA) according to the manufacturer’s protocol. To distinguish between SMN∆7 mice and their control littermates, the following primers were used: wild-type forward, 5′-CTCCGGGATATTGGGATTG-3′; wild-type reverse 5′-TTTCTTCTGGCTGTGCCTTT-3′; and SMA reverse 5′-GGTAACGCCAGGGTTTTCC-3′. PCR products were resolved by electrophoresis on a 0.8% agarose gel stained with SYBR Safe DNA stain (Molecular Probes, Eugene, OR, USA) and run at 100 V.

The SMN-ASO was synthesized and purified by Integrated DNA Technologies (IDT, Coralville, IA, USA) as previously described by Hua et al. [52,80]. The SMN-ASO targets the 10–27 nucleotide sequence (5′-TCACTTTCATAATGCTGG-3′) in intron 7 of the *SMN2* gene. All nucleotides were modified with 2′-O-(2-methoxyethyl) groups and linked via phosphorothioate (P-thio) backbones. Additionally, all cytosine and thymine residues were 5-methylated to enhance thermal stability. The complete modified sequence is: 2′-O-(2-methoxyethyl)(3′-5′)(P-thio)(5mT-5mC-A-5mC-5mT-5mT-5mT-5mC-A-5mT-A-A-5mT-G-5mC-5mT-G-G). For administration, SMN-ASO was dissolved in 0.9% NaCl.

Animals were assigned to different experimental groups: control mice and SMNΔ7 mice, which were randomly allocated to one of the following conditions: untreated, ICV treated with SMN-ASO, or SC treated with SMN-ASO. For ICV treatment, a single injection of SMN-ASO (6.5 mg/kg body weight) was administered into the lateral ventricle of P1 SMNΔ7 mice as previously described [81]. Briefly, injections were performed under cryo-anesthesia using a 10 µL Hamilton 701 syringe (Teknokroma Analítica, Sant Cugat del Vallès, Barcelona, Spain, Cat. # HA-80308) fitted with a customized 32-gauge, 10 mm-long, point style 4 needle. For SC treatment, SMN-ASO was administered at 50 mg/kg body weight/day into the interscapular region of SMNΔ7 mice on P1, P3, P5, and P7 [76]. SC injections were performed using a 50 µL Hamilton 705N syringe (Teknokroma Analítica, cat. # HA-80508) fitted with a customized 27-gauge, 12 mm-long, point style 4–30° needle. For control injections, vehicle solution (saline) was administered to a group of control and SMN∆7 mice. Mice that received either ICV or SC injections of vehicle solution showed no significant differences in lifespan or phenotype compared to non-injected littermates, indicating that vehicle administration via either route was well tolerated and had no adverse effects on the animals.

### 4.2. Mouse Motor Behavioral Analysis

To evaluate disease progression, mice were weighed and examined for the presence of specific disease-related signs and/or symptoms every two days. Motor abilities were assessed using two tests. The righting reflex test, which evaluates overall body strength and motor coordination [82], was performed every two days from P0 to P14. Briefly, pups were placed on their backs on a flat plastic surface, and the time to right themselves was recorded within a 30 s observation window. From P16 onwards, the inverted mesh grip test was conducted to measure forelimb and hindlimb strength and fatigue according to TREAT-NMD Neuromuscular Network standard operating procedure DMD_M.2.1.005 with minor modifications. Mice were suspended from an aluminum mesh grip with 0.25 cm^2^ holes, and latency to fall was recorded over a maximum period of 30 s [54]. This test was performed every 2 days from P16 to P30, every 6 days from P36 to P60, and every 12 days thereafter (Supplementary Figure S6). In each test, each mouse was examined three times, with a minimum of 3 min rest between each trial. Only the best performance from the three trials was included in the subsequent analysis. Weaning of SMN-ASO-treated SMN∆7 mice was postponed until 30 days after birth. After weaning, Hydrogel^®^ (ClearH_2_O, Westbrook, ME, USA) and standard laboratory chow softened with water were provided inside the cage to facilitate hydration and feeding.

### 4.3. Mouse Tissue Sample Collection

Thymuses and spleens were collected from SMNΔ7 and control mice at early symptomatic (P7) and late symptomatic (P14) stages of disease under anesthesia induced by intraperitoneal injection of ketamine (100 mg/kg) and xylazine (10 mg/kg). For histological and immunohistochemical analyses, animals were intracardially perfused with saline solution followed by 4% paraformaldehyde (PFA) in 0.1 M phosphate buffer (PB), pH 7.4. Following rapid dissection, organs were post-fixed overnight in the same fixative, then weighed and processed for either paraffin embedding or cryosectioning. For flow cytometry and reverse transcription-quantitative polymerase chain reaction (RT-qPCR), organs from non-perfused mice were immediately placed in ice-cold flow cytometry buffer (phosphate-buffered saline [PBS] with 5% fetal bovine serum [FBS]) or snap-frozen in liquid nitrogen, respectively. Spinal cords were rapidly dissected by carefully separating the tissue from the vertebral column, snap-frozen in liquid nitrogen, and stored at −80 °C until further processing for RT-qPCR analyses.

### 4.4. SMA Patient Tissue Samples

SMA cases for the post-mortem study were identified at the Pathology Department of Vall d’Hebron University Hospital (Barcelona, Spain). In all instances, macroscopic examination followed established protocols, and tissue was fixed in 10% buffered formalin for 1–2 weeks. SMA diagnosis was genetically confirmed by bi-allelic absence of the *SMN1* gene and the presence of one or two *SMN2* copies, as previously described [83]. All individuals had a documented family history of severe SMA. The study was approved by the Clinical Investigation Ethics Committee of Vall d’Hebron University Hospital (PR[AMI]458/2021), and written informed consent was obtained from all parents. Fetal material was collected following legally approved elective pregnancy interruption (Supplementary Figure S5A). An age- or gestational age–matched control cohort (*n* = 8) was obtained through the Pathology Department Biobank and the Genetics Department sample collection (C-00354). Post-mortem procedures for controls were identical to those applied to the SMA group.

### 4.5. Histological and Immunohistochemical Analyses of Mouse Tissue Samples

Mouse tissue samples were processed either for paraffin embedding following standard protocols or for immunohistochemical analysis on frozen sections. Paraffin-embedded tissues were sectioned at 5 µm, mounted on slides, and gradually rehydrated through an ethanol series. Sections were then either stained with H&E or processed for peroxidase-based immunohistochemistry. For cryosectioning, tissues were cryoprotected in 30% sucrose in 0.1 M PB, embedded in Tissue Freezing Medium (TFM; Triangle Biomedical Sciences, Durham, NC, USA), and frozen. Cryostat sections (10 µm thick) were obtained and stored at −80 °C until further processing for immunofluorescence analysis.

For immunoperoxidase staining, deparaffinized sections were rinsed in PBS containing 0.1% Triton X-100 for 30 min, followed by incubation in 1% H_2_O_2_ peroxide in PBS for 1 h at room temperature (RT) to quench endogenous peroxidase activity. After several washes in PBS–0.1% Triton X-100, sections were blocked with normal goat serum and incubated overnight at 4 °C with one of the following primary antibodies: rabbit monoclonal anti-CD3Ɛ (1:200; Cell Signaling Technology, Danvers, MA, USA; cat. #78588) for T lymphocytes, or rabbit monoclonal anti-CD20 (1:800; Cell Signaling Technology; cat. #70168) for B lymphocytes. Following primary antibody incubation, sections were rinsed in PBS and incubated for 1 h at RT with a biotinylated goat anti-rabbit IgG secondary antibody (1:100; Vector Laboratories, Burlingame, CA, USA; cat. #BA-100-1.5). After additional PBS washes, the VECTASTAIN^®^ ABC reagent (1:100; Vector Laboratories) was applied for 1 h at RT. Immunoreactivity was visualized using a 3,3′-diaminobenzidine (DAB)/hydrogen peroxide chromogenic reaction. Finally, sections were counterstained with Mayer’s hematoxylin, dehydrated, and mounted using DPX mounting medium. Mounted sections were imaged using an Olympus BX50 optical microscope (Olympus, Hamburg, Germany) equipped with a DXM 1200 Nikon digital camera (Nikon, Tokyo, Japan).

For immunofluorescence analyses, cryostat sections were permeabilized in PBS containing 0.1% Triton X-100 for 30 min, followed by blocking with 10% normal goat serum in PBS for 1 h at RT. Sections were then incubated overnight at 4 °C with the primary antibodies, mouse monoclonal anti-CD31 (1:100; BioLegend, San Diego, CA, USA; cat. #102402) or rabbit polyclonal anti-F4/80 (1:200; Proteintech, Rosemont, IL, USA; cat. #28463-1-AP). After PBS rinses, sections were incubated for 1 h at RT with the appropriate fluorophore-conjugated secondary antibodies (1:500), including anti-rat Alexa Fluor 488, anti-mouse Cy3, and anti-rabbit Alexa Fluor 647 (Jackson ImmunoResearch Laboratories, West Grove, PA, USA; cat. #712-546-153, #715-166-151, and #711-606-152, respectively). Nuclei were counterstained with DAPI (1:100; Sigma-Aldrich, St. Louis, MO, USA). Sections were mounted in antifade medium composed of 0.1 M Tris-HCl buffer (pH 8.5), 20% glycerol, 10% Mowiol (Sigma-Aldrich; cat. #32459-0), and 0.1% 1,4-diazabicyclo[2.2.2]octane (DABCO; Sigma-Aldrich; cat. #D-2522).

Immunohistochemical controls were performed by omitting the primary antibodies in parallel-processed sections, which showed no detectable staining. To ensure consistency across experimental conditions, tissue sections from different animals and groups were processed in parallel for both immunohistochemistry and subsequent imaging.

Tissue sections processed for immunofluorescence were examined using a FluoView FV-1000 confocal microscope (Olympus). When necessary, the mosaic function was used to stitch individual high-magnification images (60×) into larger composite images encompassing the entire sample. Multiple Z-planes were acquired to generate a three-dimensional mosaic of adjacent image stacks. Digital images were subsequently processed and analyzed using ImageJ 1.54p software (Fiji distribution; U.S. National Institutes of Health [NIH], Bethesda, MD, USA).

### 4.6. Histological and Immunohistochemical Analyses of Human Tissue Samples

Tissue samples from human thymuses and spleens were embedded in paraffin. Sections were stained with H&E or processed for immunohistochemical analysis. Immunohistochemical procedures were performed using the Benchmark Ultra staining system (Ventana Medical Systems, Tucson, AZ, USA) in combination with the ultraView Universal DAB Detection Kit (cat. #760-500). Deparaffinization was performed using EZ prep™ solution at 75 °C for 8 min (cat. #950-102). Antigen retrieval was carried out with Cell Conditioning Solution CC1, a Tris-EDTA-based buffer (pH 7.8, cat. #950-124). Endogenous peroxidase activity was quenched using 3% H_2_O_2_, followed by rinsing with Reaction Buffer, a Tris-based buffer (pH 7.6–7.8, cat. #950-300). All steps were accomplished automatically by the staining module.

Immunoperoxidase staining was performed on paraffin-embedded sections using the following primary antibodies: rabbit monoclonal anti-CD3 (clone 2GV6; 0.4 μg/mL; Ventana Medical Systems, cat. #790-4341) and mouse monoclonal anti-CD20 (clone L26; 0.3 μg/mL; Ventana Medical Systems, cat. #760-2531). Slides were incubated with the primary antibodies for 40 min at RT. Following incubation, sections were washed and treated with a horseradish peroxidase (HRP)-conjugated secondary antibody cocktail (anti-DIG HRP Multimer; Roche, Basel, Switzerland; cat. #760-4822), which included goat anti-mouse and goat anti-rabbit antibodies. Immunoreactivity was visualized using DAB chromogen. Sections were counterstained with hematoxylin and Bluing Reagent (Ventana Medical Systems, cat. #760-2021 and #760-2037, respectively). Upon completion of the automated staining protocol, slides were washed, dehydrated, and mounted with DPX mounting medium.

Immunofluorescence staining was performed on selected sections. Slides were deparaffinized in xylene, fixed in 100% ethanol for 10 min, rehydrated, and washed in Tris-buffered saline (TBS; pH 7.6). Antigen retrieval was conducted by microwave heating in a citric acid monohydrate buffer (pH 6.0). After additional washes in TBS, sections were blocked for 1 h at RT in 5% bovine serum albumin (BSA) diluted in TBS. Primary antibodies were diluted in 2.5% BSA-TBS and incubated overnight at 4 °C. The antibodies used were rabbit recombinant monoclonal anti-HLA-E (for MHC-I, 1:100; Abcam; cat. #EPR25300-104) and rabbit recombinant monoclonal anti-HLA-DR (for MHC-II; 1:100; Abcam, Cambridge, UK; cat. #EPR3692). After rinsing, sections were incubated for 1 h at RT with the following secondary antibodies, also diluted in 2.5% BSA-TBS: donkey anti-rabbit IgG Alexa Fluor™ 488 (1:1000; Thermo Fisher Scientific) and goat anti-mouse IgG Alexa Fluor™ 568 (1:1000; Thermo Fisher Scientific). Finally, slides were washed and mounted with ProLong™ Gold Antifade Mountant containing DAPI (Invitrogen–Thermo Fisher Scientific, Waltham, MA, USA).

Whole-slide digital images were acquired using the PANNORAMIC 250 Flash III scanner and SlideScanner software v2.3 (3DHISTECH, Budapest, Hungary). Image analysis was performed using CaseViewer software v2.4 (3DHISTECH). Two sections per organ were examined, and representative 10× fields of view (FOVs) from defined anatomical regions were selected and exported as GIF images. These images were subsequently analyzed using ImageJ software (Fiji distribution; U.S. National Institutes of Health [NIH], Bethesda, MD, USA). For immunohistochemical analysis, each image was converted to 8-bit grayscale before quantification. Results represent data from a minimum of *n* = 10 sections per sample.

### 4.7. Flow Cytometry

Thymuses and spleens from non-perfused mice were mechanically dissociated using the frosted ends of two sterile glass slides to obtain single-cell suspensions. For bone marrow isolation, hindlimbs were dissected, and the epiphyses of the femurs and tibias were removed. A 30G needle was inserted into the bone shafts, and flow cytometry buffer was flushed through to collect the marrow cell suspension. Cell suspensions from samples were centrifuged at 120× *g* for 5 min, and supernatants were discarded. Pellets were resuspended in 900 μL of sterile water to induce erythrocyte lysis by osmotic shock, and the reaction was promptly stopped by restoring osmolarity with 100 μL of 10× PBS. Cells were then counted using a Neubauer chamber, and 2 × 10^6^ cells were aliquoted into each tube for flow cytometry analysis. Before staining, samples were incubated for 20 min at RT with blocking solution (Mouse Seroblock FcR, Bio-Rad, Hercules, CA, USA; cat. #BUF041A, diluted 1:100 in flow cytometry buffer). After washing with flow cytometry buffer, cells were incubated for 10 min at RT in the dark with primary fluorochrome-conjugated antibodies (all from BD Biosciences, Franklin Lakes, NJ, USA) diluted 1:100 in flow cytometry buffer. The following antibodies were used: rat anti-mouse CD3-APC-Cy™7 (cat. #560590); rat anti-mouse CD19-FITC (cat. #561740); rat anti-mouse CD4-PE (cat. #553730); rat anti-mouse CD8-BV421 (cat. #563898); rat anti-mouse CD117-APC (cat. #553356); rat anti-mouse CD127-BV421 (cat. #562959); rat anti-mouse CD45R-V500 (cat. #561226); rat anti-mouse CD11b-PE-Cy™7 (cat. #562222); and F4/80-Alexa Fluor^®^ 488 (cat. #564227). After washing, cells were incubated with AnxV (BD Biosciences, cat. #550474) and 7-AAD (Invitrogen, Waltham, MA, USA; cat. #A1310) for apoptosis analysis. Specifically, 5 μL of AnxV and 2.5 μL of a 1:10 dilution of 7-AAD were added to 100 μL of AnxV binding buffer (BD Biosciences, cat. #556454) and incubated for 15 min at RT in the dark. Samples were then centrifuged and resuspended in flow cytometry buffer for acquisition using a FACS Canto II flow cytometer (BD Biosciences). Data were analyzed using FACSDiva software, version 6.1.3 (BD Biosciences).

### 4.8. Reverse Transcription-Quantitative Polymerase Chain Reaction (RT-qPCR)

Total RNA was extracted from the spleens and thymuses of P14 mice using the NucleoSpin^®^ RNA isolation kit (Macherey-Nagel, Düren, Germany; cat. #740955) following the manufacturer’s instructions. RNA concentrations were measured using a NanoPhotometer spectrophotometer (Implen N60-Touch). Reverse transcription and quantitative PCR (qPCR) amplification were performed simultaneously using the iTaq™ Universal SYBR^®^ Green One-Step kit (Bio-Rad).

The following primers were used: *hSMN2* ex6/ex6 (forward 5′-ATAATTCCCCCACCACCTCC-3′; reverse 5′-TAGCCAGTATGATAGCCACTCATGTA-3′); *hSMN2* ex6/ex7 (forward 5′-TACATGAGTGGCTATCATACTGGCTA-3′; reverse 5′-AATGTGAGCACCTTCCTTCTTTTT-3′); *hSMN2* ex5-6/ex6-8 (forward 5′-TTCCTTCTGGACCACCAATAA-3′; reverse 5′-TGCTCTATGCCAGCATTTCCATAT-3′); *GAPDH* (forward 5′-AGGGCTGCCTTCTCTTGTGAC-3′; reverse 5′-TGGGTAGAATCATACTGGAACATGTAG-3′; *mIL6* (forward 5′-ACCAGAGGAAATTTTCAATAGGC-3′; reverse 5′-TGATGCACTTGCAGAAAACA-3′) and *TNFα* (forward 5′-CAGGCGGTGCCTATGTCTC-3′; reverse 5′-CGATCACCCCGAAGTTCAGTAG-3′).

Each qPCR reaction contained 100 ng of RNA, iTaq Universal SYBR^®^ Green reaction mix (2×; Bio-Rad, Hercules, CA, USA; cat. #172-5150), iScript reverse transcriptase (Bio-Rad), RNase/DNase-free water, and primers at a final concentration of 1 µM, in a total reaction volume of 10 µL. Amplifications were performed using the QuantStudio™ 6 Pro system (Proteomic and Genomic Service, IRBLleida). Gene expression data were analyzed using the ThermoFisher Connect Platform (Thermo Fisher Scientific). Expression levels were normalized to *GAPDH*, and relative quantification was calculated using the 2^−ΔΔCt^ method to determine fold changes in target gene expression.

### 4.9. Statistical Analysis

Data are presented as mean ± standard error of the mean (SEM). Statistical analyses were performed using either one-way or two-way analysis of variance (ANOVA), followed by Tukey’s post hoc test, or a two-tailed Student’s *t*-test for comparisons between two groups. Prior to statistical analysis, data distributions were assessed for normality using the Shapiro–Wilk test. Survival curves were compared using the Gehan–Breslow–Wilcoxon test. A *p*-value ≤ 0.05 was considered statistically significant. All statistical analyses and data visualizations were performed using GraphPad Prism 8 (GraphPad Software Inc., San Diego, CA, USA).

## 5. Conclusions

In conclusion, our study demonstrates that early systemic administration of SMN-targeting ASOs can prevent lymphoid organ abnormalities in a severe SMA mouse model, supporting a direct requirement for SMN in peripheral immune system development. Although CNS-targeted approaches such as ICV delivery were also effective in mice, likely due to immaturity of the neonatal BBB, this route is unlikely to ensure comparable peripheral exposure in humans, in whom postnatal BBB maturation limits peripheral drug distribution. These findings highlight the translational relevance of systemic SMN restoration and support the development of therapeutic strategies with broad biodistribution to address both central and peripheral pathologies. Ultimately, recognizing and targeting the multisystemic nature of SMA will be essential to optimize long-term outcomes in the growing population of treated patients.

## Data Availability

The data that support the findings of this study are available from the corresponding author upon reasonable request.

## Supplementary Materials

The following supporting information can be downloaded at: https://www.mdpi.com/article/10.3390/ijms27031274/s1.

## Abbreviations

7-AAD	7-aminoactinomycin D
AnxV	annexin V
ASO	antisense oligonucleotide
BBB	blood–brain barrier
BSA	bovine serum albumin
CNS	central nervous system
DN	double negative
DP	double positive
EMA	European Medicines Agency
FACS	fluorescence-activated cell sorting
FBS	fetal bovine serum
FDA	Food and Drug Administration
FL	full-length
H&E	hematoxylin and eosin
HSC	hematopoietic stem cell
ICV	intracerebroventricular
IL-6	interleukin-6
MN	motor neuron
P	postnatal day
PALS	periarteriolar lymphoid sheath
PB	phosphate buffer
PBS	phosphate-buffered saline
PCR	polymerase chain reaction
PFA	paraformaldehyde
RT	room temperature
RT-qPCR	reverse transcription-quantitative polymerase chain reaction
SC	subcutaneous
SEM	standard error of the mean
SMA	spinal muscular atrophy
SMN	survival motor neuron
SP	single positive
TNF-α	tumor necrosis factor-α
