# Supplementary material for: Lymphoid Organ Architecture and Hematopoiesis Disruption in Spinal Muscular Atrophy: Therapeutic Rescue by SMN Restoration
Source: Int J Mol Sci. 2026 Jan 27;27(3):1274. doi: 10.3390/ijms27031274 (PMC12897893; doi:10.3390/ijms27031274)
Supplement: Supplementary file 1 [file ijms-27-01274-s001.zip › ijms-4050976-supplementary.pdf]

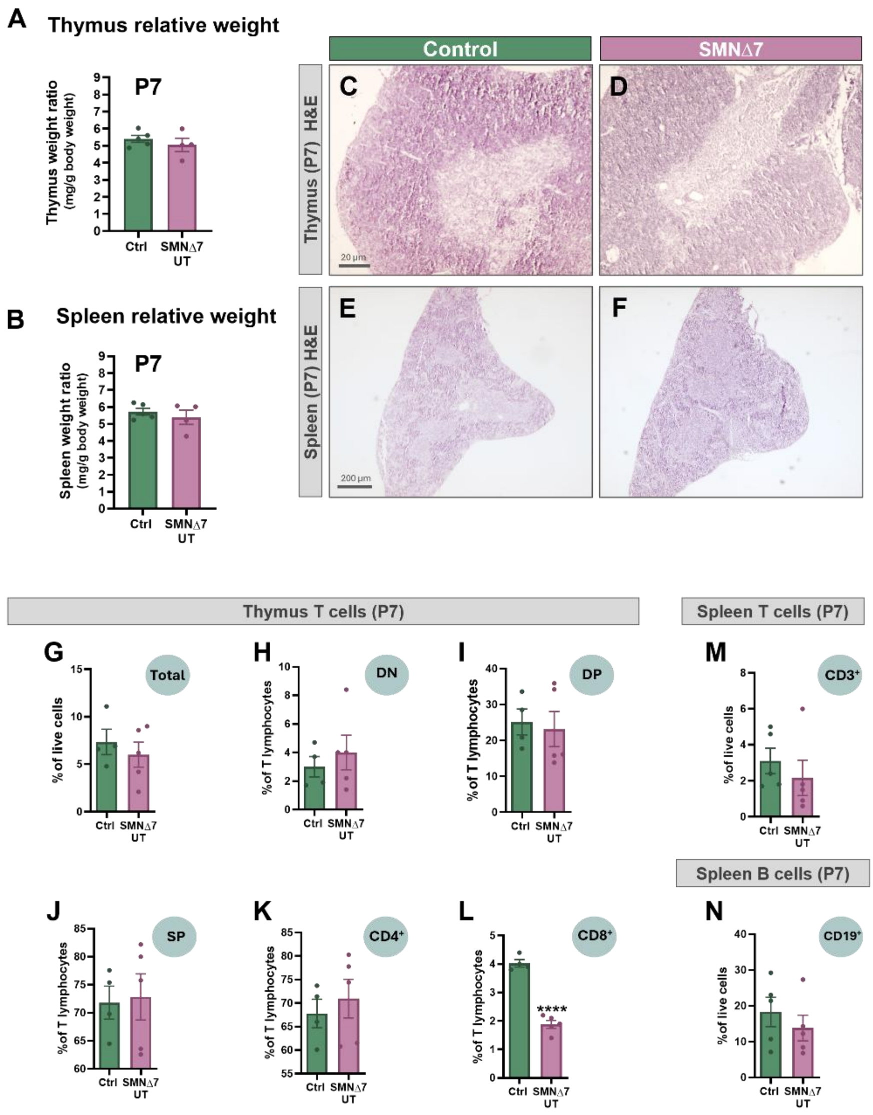

Supplementary Figure S1. Lymphoid organs appear macroscopically and histologically unaltered in SMNΔ7 mice at the pre-symptomatic stage (P7). **A,B)** Quantification of thymus (**A**) and spleen (**B**) weight normalized to total body weight in P7 control (Ctrl), untreated (UT)-SMNΔ7, and SMN-ASO-treated SMNΔ7 mice via intracerebroventricular (ICV) or subcutaneous (SC) injection (n = 4-5 mice per group). Statistical analysis was performed using one-way ANOVA followed by Tukey's post hoc test. Black asterisk indicates statistically significant difference between SMNΔ7+ICV-ASO and SMNΔ7+SC-ASO groups (*$p < 0.05$). **C–F)** Representative H&E-stained thymus (**C,D**) and spleen (**E,F**) sections from P7 control and untreated SMNΔ7 mice. At this age, the splenic white and red pulp are not yet clearly distinguishable. Scale bars: in **C** = 20 µm (applies to **D**) and in **E** = 200 µm (applies to **F**). **G-N)** At P7, flow cytometry showed comparable thymic and splenic immune cell profiles between control (Ctrl) and untreated (UT) SMNΔ7 mice, except for a reduced $CD8^+$ population in the thymus of UT mutants. Thymocytes and splenocytes from P7 control (Ctrl) and SMNΔ7 untreated (UT) mice were surface-labeled with CD3 (T cell marker) and CD19 (B cell marker). In addition, thymic T cells ($CD3^+$) were further analyzed based on CD4 and CD8 surface expression. Four distinct T-cell subsets were identified: double negative (DN; $CD4^-CD8^-$), double positive (DP; $CD4^+CD8^+$), CD4 single positive ($CD4^+CD8^-$), and CD8 single positive ($CD4^-CD8^+$), as shown. **G)** Percentage of T cells in the thymus ($CD3^+$) relative to total live thymocytes. **H–L)** Percentages of thymic T-cell subsets based on CD4/CD8 expression, including the combined single positive (SP; $CD4^+$ and $CD8^+$) population (percentages are relative to total T cells). **M–N)** Percentages of T cells ($CD3^+$) (**M**) and B cells ($CD19^+$) (**N**) in the spleen relative to total live splenocytes. Asterisks indicate significant differences between UT SMNΔ7 and control mice (*$p < 0.05$; **$p < 0.01$; ***$p < 0.001$). All data in graphs are expressed as mean ± SEM.

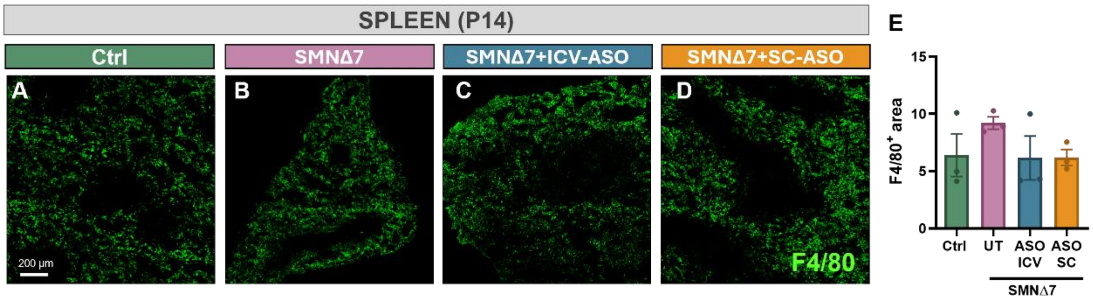

Supplementary Figure S2. SMNΔ7 spleens exhibit a modest increase in the density of resident macrophages, which is prevented by ICV or SC administered SMN-ASO. **A–D)** Representative splenic sections from P14 control (**A**), untreated (UT) SMNΔ7 (**B**), ICV-ASO–treated (**C**), and SC-ASO–treated (**D**) mice immunolabeled with anti-F4/80 antibody, a marker of resident splenic macrophages. Scale bar in **A** = 200 µm (applies to **B**-**D**). **E)** Quantification of F4/80^{+} area relative to the total spleen section (n = 3 mice per group). Data are presented as mean ± SEM. Statistical analysis was assessed by one-way ANOVA followed by Tukey's post hoc test.

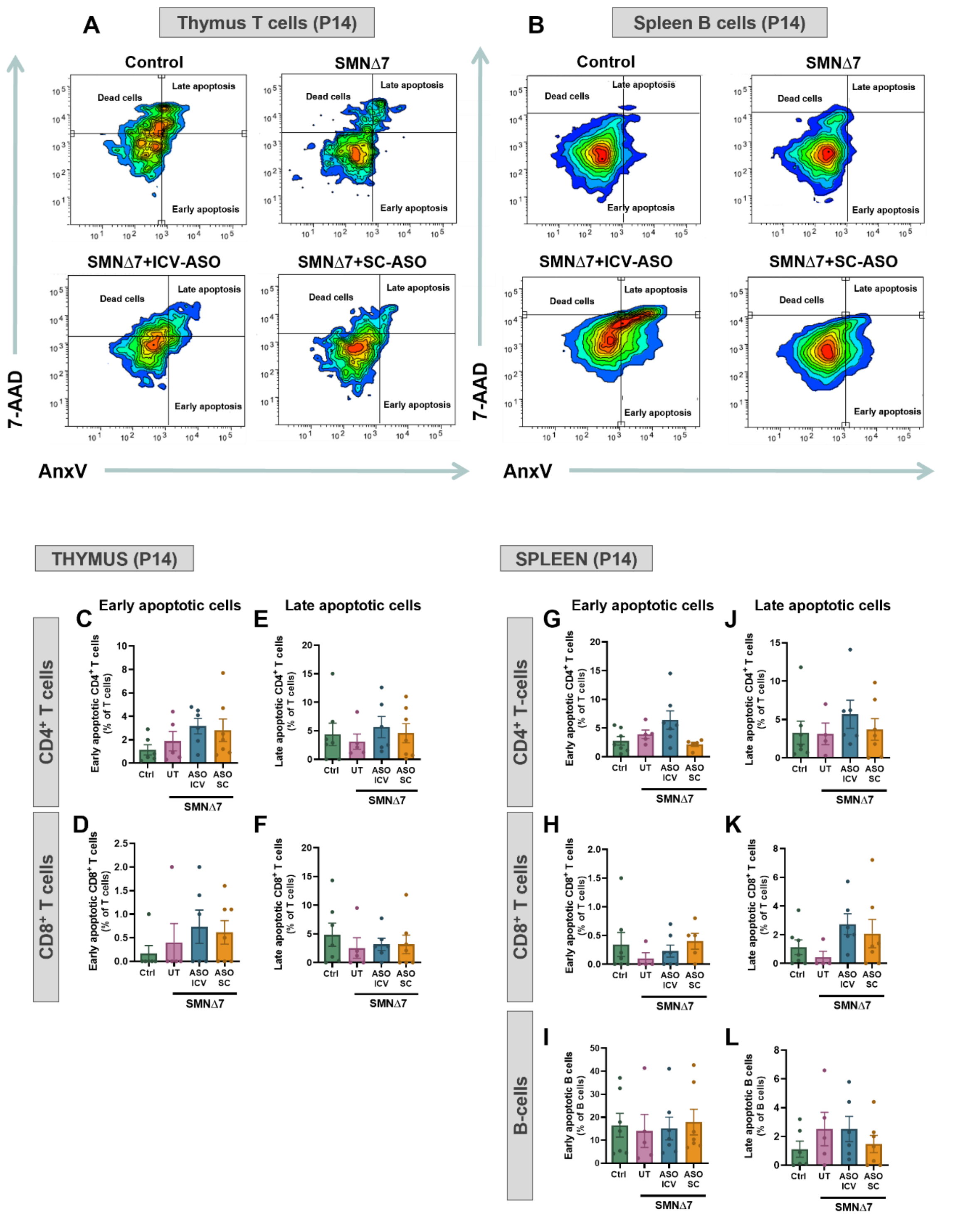

Supplementary Figure S3. Early and late apoptosis analysis based on the expression of Annexin V (AnxV) and 7-AAD in B cells, and CD4$^+$ and CD8$^+$ T cells from the thymus and spleen of P14 mice. **A-B)** Representative FACS plots of thymus T cells (**A**) and spleen B cells (**B**) showing staining with AnxV and 7-AAD. Four gates distinguish live cells (AnxV$^-$7-AAD$^-$), early apoptotic cells (AnxV$^+$7-AAD$^-$), late apoptotic cells (Anx5$^+$7-AAD$^+$), and dead cells (AnxV$^-$7-AAD$^+$). **C–L)** Percentages of early and late apoptotic cells in thymic (**C-F**) and splenic (**G-L**) CD4$^+$ T cells, and CD8$^+$ T cells, and B cells (as indicated), gated as shown in **A** and **B**. Cells were obtained from thymuses and spleens of control (Ctrl), untreated SMNΔ7 (UT-SMNΔ7), ICV-ASO-treated, and SC-ASO-treated mice at P14. No significant differences were found between the four experimental groups. Data are presented as mean ± SEM. Statistical analysis was assessed using one-way ANOVA followed by Tukey's post hoc test**.**

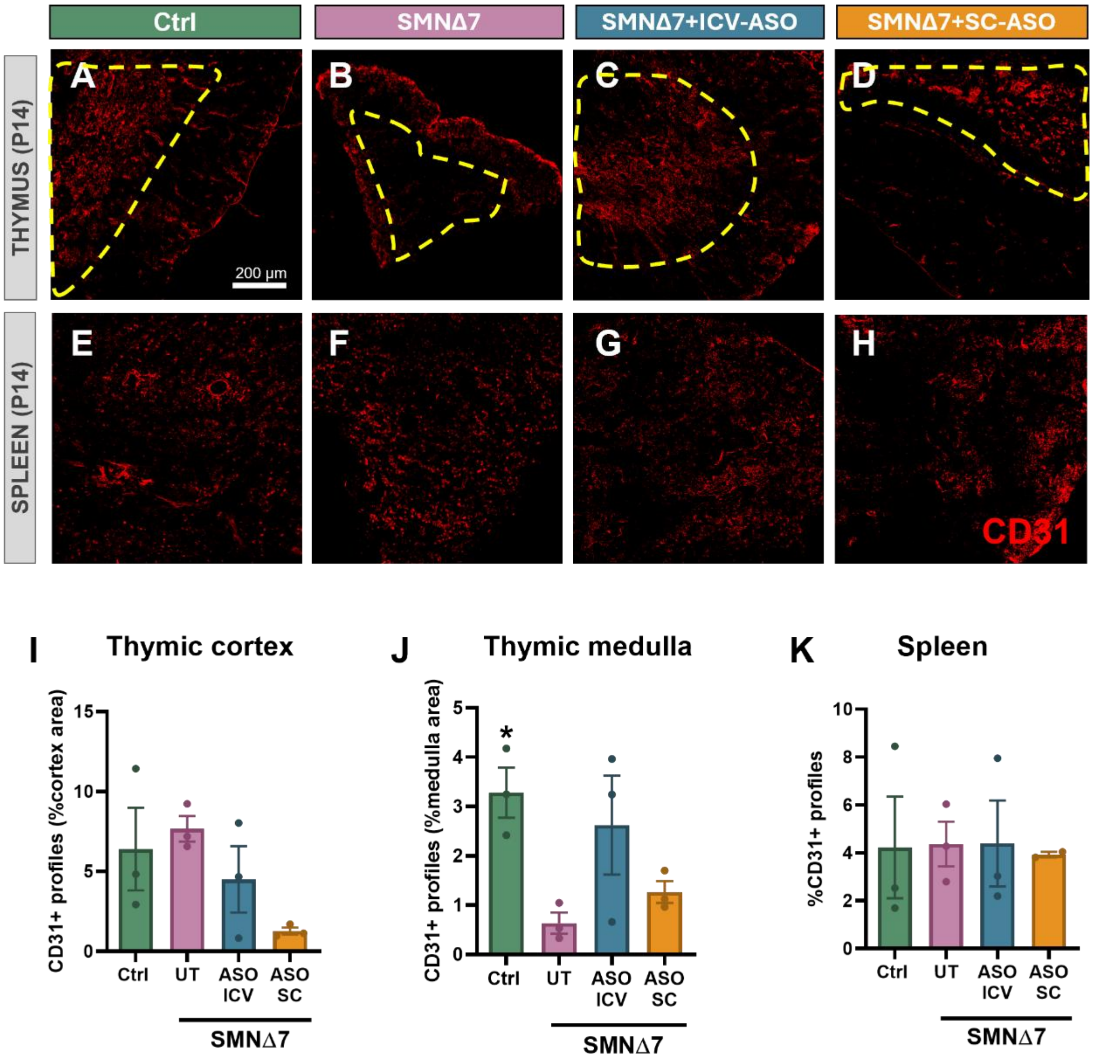

Supplementary Figure S4. SMNΔ7 mice show no significant changes in splenic vascularization but exhibit vasculature defects in the thymic medulla. **A-H)** Representative immunofluorescence images for CD31, a marker of endothelial cells, in sections taken from thymuses (**A-D**) and spleens (**E-D**) of P14 control, SMNΔ7 untreated, ICV-ASO treated, and SC-ASO treated mice, as indicated in panels. The thymic medulla is delimited by a yellow dotted line. Scale bar in **A** = 200 μm (applies to **B-H**). **I-K)** Quantification of the percentage of $CD31^{+}$ immunostaining in the thymic cortex (**I**) and medulla (**J**), and total spleen (**K**) relative to total areas of respective sections (n = 3 mice per group). Data in the graph are expressed as mean ± SEM. Significant differences were assessed using one-way ANOVA followed by Tukey's post hoc test; *$p < 0.05$ of untreated SMNΔ7 vs. control, SMNΔ7+ICV-ASO, and SMNΔ7+SC-ASO.

A

	Non-SMA (Ctrl) sample size	Non-SMA gestational age	SMA sample size	SMA gestational age	SMA type	Number of *SMN2* copies
Prenatal	2	13 weeks	2	13 weeks	1	2
	2	18 weeks	1	18 weeks	1	2
	0	21 weeks	1	21 weeks	0	1
Perinatal	0	36 weeks	1	36 weeks	0	1
	2	1 month	1	1 month	0	1
Postnatal	2	8 months	1	8 months	1	2

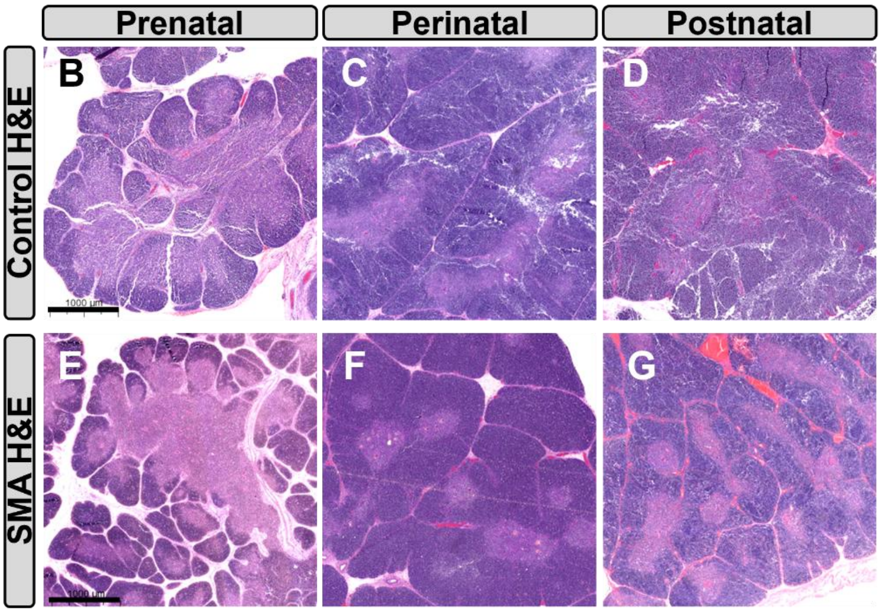

H

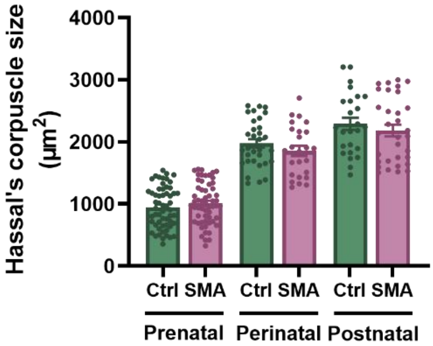

Supplementary Figure S5. Thymus histology in control and SMA fetuses and patients. **A)** Summary of postmortem human tissue samples included in this study. All samples were obtained from clinically and genetically confirmed SMA and non-SMA (control) fetuses and patients, following informed consent and ethical approval (see Methods for details). **B–F)** Representative H&E-stained thymic sections at prenatal, perinatal, and postnatal stages shown at higher magnification. Scale bar in **B** = 1000 µm (applies to **C–F**). **H)** Quantification of the overall size (µm²) of Hassall's corpuscles in control and SMA thymic samples at different developmental stages. Data are presented as mean ± SEM. No significant differences were detected between groups at any developmental stage (one-way ANOVA followed by Tukey's post hoc test). Sample sizes are listed in **A**.

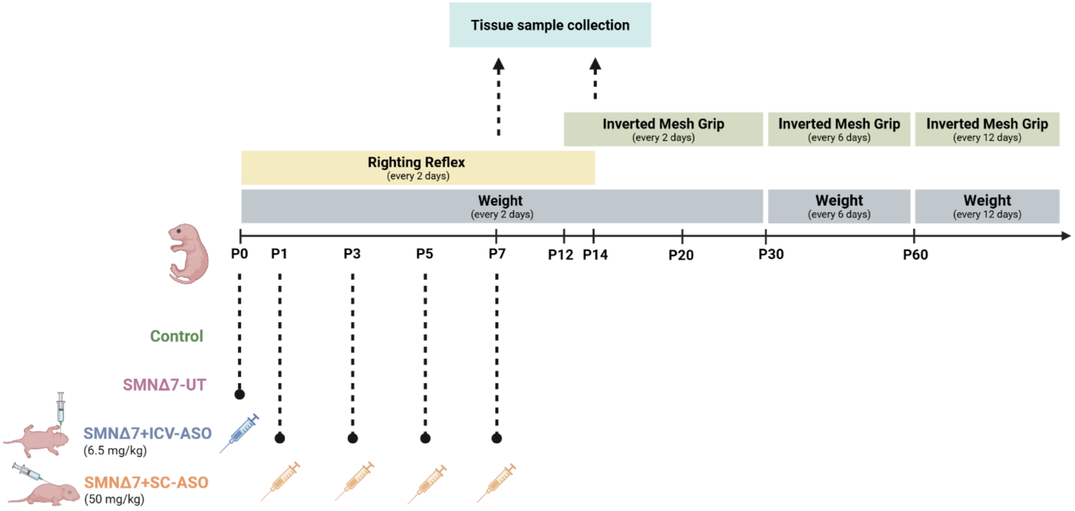

Supplementary Figure S6. Experimental design to evaluate the impact of ASO treatment on lymphoid organ pathology associated with SMA. Two delivery strategies were used: a single intracerebroventricular injection on P0 (ICV-ASO) or several subcutaneous injections (SC-ASO) on P1, P3, P5, and P7.
